# The Adaptative Modulation of the Phosphinito–Phosphinous Acid Ligand: Computational Illustration Through Palladium-Catalyzed Alcohol Oxidation

**DOI:** 10.3390/molecules29214999

**Published:** 2024-10-22

**Authors:** Romain Membrat, Tété Etonam Kondo, Alexis Agostini, Alexandre Vasseur, Paola Nava, Laurent Giordano, Alexandre Martinez, Didier Nuel, Stéphane Humbel

**Affiliations:** 1 Aix Marseille Univ, CNRS, Centrale Med, ISM2, Marseille, France; romainmembrat@gmail.com (R.M.); alexandre.vasseur@univ-lorraine.fr (A.V.); paola.nava@univ-amu.fr (P.N.); laurent.giordano@centrale-marseille.fr (L.G.); alexandre.martinez@centrale-marseille.fr (A.M.); 2 Université de Lorraine, CNRS, L2CM, F-54000 Nancy, France

**Keywords:** DFT, reaction path, alcohol oxidation, Pd catalysis, ligand

## Abstract

The phosphinito–phosphinous acid ligand (PAP) is a singular bidentate-like self-assembled ligand exhibiting dissymmetric but interchangeable electronic properties. This unusual structure has been used for the generation of active palladium hydride through alcohol oxidation. In this paper, we report the first theoretical highlight of the adaptative modulation ability of this ligand within a direct H-abstraction path for Pd and Pt catalyzed alcohol oxidation. A reaction forces study revealed rearrangements in the ligand self-assembling system triggered by a simple proton shift to promote the metal hydride generation via concerted six-center mechanism. We unveil here the peculiar behavior of the phosphinito–phosphinous acid ligand in this catalysis.

## 1. Introduction

**Figure 1:**
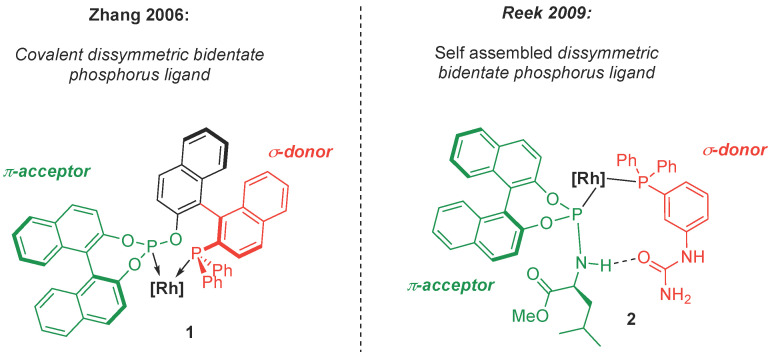
Covalent and H-bond assisted self-assembled bidentate di-phosphorus ligands.

In homogenous catalysis, the ligands of a reactive metal center have an enormous impact on the reactivity. The fine tuning of the ligands is thus desirable to achieve the synthesis of highly functionalized stereospecific targets with a straightforward approach [1]. In this context, bidentate diphosphines are interesting ligands, because the rigidity due to the chelate effect can contribute to the stereocontrol [2,3,4,5]. Dissymmetric di-organophosphorus ligands bearing both strong σ-donor and π-acceptor units (Figure 1(1)) proved to be particularly efficient in enantioselective hydroformylations [6].

**Scheme 1:**
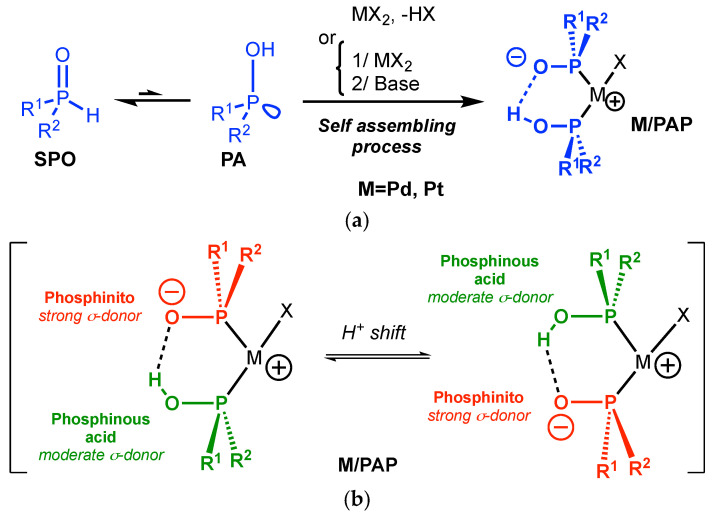
(**a**) The self-assembled phosphinito–phosphinous acid ligand/metal complex (M/PAP). (**b**) The dissymmetric nature of the M/PAP catalyst.

However, the introduction of purely covalent spacing groups between the two donating sites requires time-consuming, tedious and stepwise synthesis. Another approach relies on the harnessing of noncovalent interactions between two monodentate organophosphorus ligands [7,8,9] to mimic the structure of a bidentate ligand through a self-assembling process as illustrated by Reek et al. in 2009 (Figure 1(2)) [10,11]. The chemistry of the phosphinito–phosphinous acid (PAP) ligand is based on this paradigm. The PAP ligand is obtained by coordination of two secondary phosphine oxides (SPOs, Scheme 1) in their phosphinous acid form (PA, Scheme 1) [12] and their self-assembling by hydrogen bonding is triggered by deprotonation (Scheme 1) [13,14,15,16,17,18,19]. M/PAP complexes have been widely used as catalysts in a broad range of reactions, as overviewed by Ackermann [20], Achard [21], and more recently by Verdaguer [22] and van Leeuwen [23]. The PAP ligand allows us to achieve high performances in CC bond formation [12,24,25].

It should be stressed that a simple proton shift in the “pincer” formed by the two phosphines (Scheme 1b) results in a switch of the electronic properties of the two phosphorus moieties [26,27]. The PAP ligand can indeed present a dissymmetric structure with one phosphinous acid (moderate σ-donor) and one phosphinito moiety (excellent σ-donor) as evidenced by our group in 2011 [28]. The presence of a single signal in ^31^P NMR for Pd/PAP complexes in CDCl_3_ reflects an equilibrium between the two forms in solution (see ESI) [16].

**Scheme 2:**
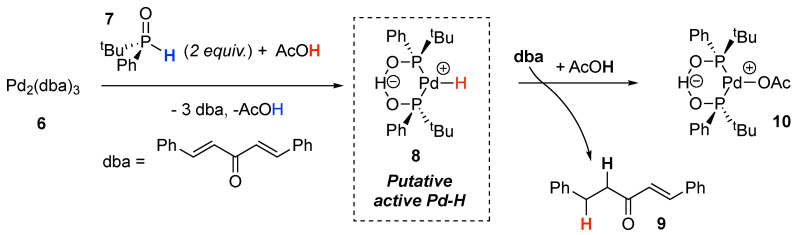
Proposed pathway for generation of active Pd-H.

The synthesis of the self-assembled Pd/PAP complex **10** from Pd_2_(dba)_3_, ^t^BuPhP(O)H **7**, and acetic acid was shown to result in the formation of monoreduced dba (dibenzylideneacetone) **9** as a by-product (Scheme 2) [29]. This experimental feature clearly indicates that the self-assembled negatively charged structure of the PAP ligand in Pd/PAP and Pt/PAP complexes enables the generation of active metal-hydride intermediates **8** from H-donors.

This property has been recently exploited through palladium and platinum catalyzed anaerobic alcohol oxidations [30,31], coordination complex synthesis [16], isomerizations [16] or one-pot oxidation–fragmentation reactions [32]. However, it is quite surprising to observe that relatively moderately hindered phosphinous acids (Cy_2_POH or Ph_2_POH) (p-cymene) are also suitable for cross-coupling procedures [33]. This result suggests a particular effect of the PAP ligand within the catalytic cycle.

**Scheme 3:**
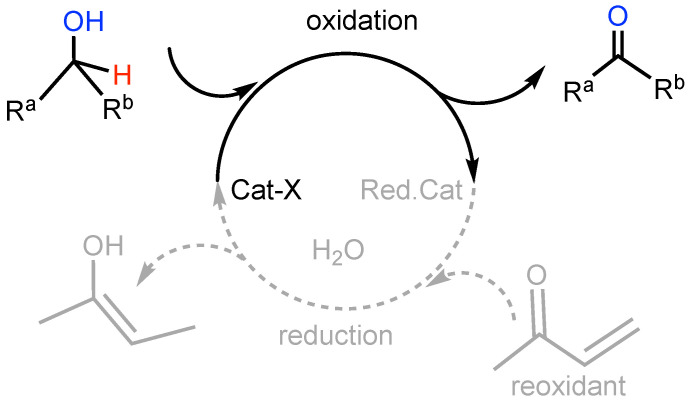
A general secondary alcohol oxidation as part of a catalytic process with a sacrificial enone reoxidant to restore the catalyst.

The anaerobic alcohol oxidation seemed well suited to clarify the behavior of the PAP ligand as a part of a catalytic cycle (Scheme 3) [34,35].

Herein, we report a mechanistic study highlighting an adaptive self-modulation of the PAP ligand during the M/PAP catalyzed alcohol oxidation. Our theoretical study focuses on the oxidation part of the catalytic cycle (Scheme 3, top).

## 2. Results

### 2.1. Experimental Study: Comparison of PAP Ligand with Classical Phosphorus Ligands

In order to know more about the role of the anionic self-assembling of the PAP ligand during the oxidation process, we ran comparative oxidation reactions of the same tetramethylpiperidin-4-ol **11**, using various structurally similar Pd and Pt catalysts (Table 1). The methyl vinyl ketone **13** acted as a sacrificial reoxidant for the catalyst. The reactions were carried out at room temperature under slightly basic conditions. The yields in tetramethylpiperidin-4-one **14** are reported in Table 1.

The best catalyst Pt/PAP complex **12a** (Table 1, entry 1) led to very good yield (74%), and the use of a similar Pd-based complex **12b** also resulted in a robust catalytic system despite a lower yield (entry 2). The replacement of **12b** by commercial catalysts **12c** or **12d** resulted in instantaneous black Pd deposit (Table 1, entries 3 and 4). In these two cases, the generated neutral Pd(II) hydride suffered from degradation by the reductive elimination of HCl. To ensure a better comparison with the PAP ligand, we replaced **12b** by the Hermann Beller catalyst **12e** with a neutral bidentate ligand bearing both ligand types L and X, with the same charge and same oxidation state as **12b**. This catalyst should lead easily to a cationic Pd-H intermediate, which is necessary for the alcohol oxidation. The obtaining of a Pd black deposit (Table 1, entry 5) clearly indicates that the PAP pincer has other characteristics in the metal chelate structure. This catalyst (**12e**) has a clear and fixed dissymmetry: a Pd-C bond on one side and a Pd-P bond on the other, while the PAP ligand (as in **12b**) can modulate the nature of the two Pd-P bonds through an inter-ligand hydrogen bonding [26,27]. Hence, this H bond seems important.

Thus, we studied the performances of the purely monodente neutral ligand system composed of two phosphinous acids (**12f**), which can feature an inter-ligand H bond as in **12b**. However, it resulted in a quick black Pt deposit after about two turn-overs, so the inter-ligand H-bond is not sufficient for the reaction to proceed smoothly. The catalyst **12g** is a derivative of **12a**, where the H-bonding proton has been replaced by a BF_2_ moiety, according to Leung’s procedure [36]. As for **12a**, **12b**, and **12e**, this catalyst can generate a neutral Pt–hydride species. It also features a clear inter-ligand bonding through Lewis Acid-Base pairings. It gives **14** in a good yield (74%, Table 1, entry 7). It should be noticed that in this case, the ^19^F NMR analysis of the crude mixture at the end of the reaction suggests that the chemical integrity of the O-BF_2_-O moiety is preserved throughout the transformation.

The contrasting results obtained here can all be attributed to the specific feature of an adaptive ligand that modulates its electronic donation ability along the reaction path. We unveiled this distinctive characteristic in a previous work on an unusual C-C bond formation [26,27]. The self-assembling of the PAP ligand seems to be robust and imperative for this alcohol oxidation reaction. In the calculations, we considered a simplified PAP ligand (PMe_2_O..H..OPMe_2_) to study the isopropanol oxidation. Such a model will be used in DFT computations to better understand the role of this versatile PAP ligand.

### 2.2. Mechanistic Computational Study

**Scheme 4:**
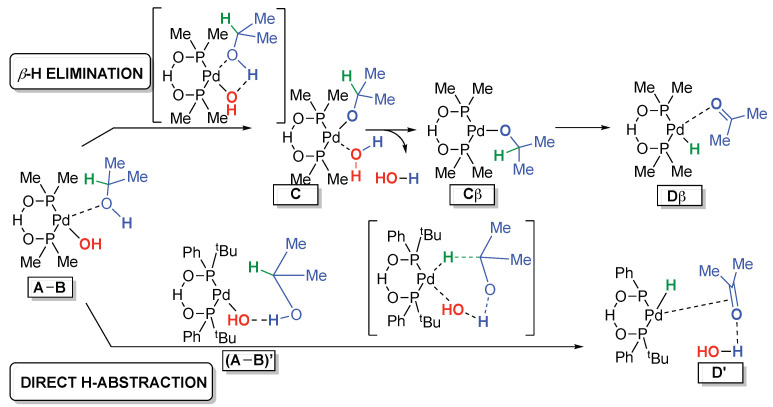
Compared pathways for the alcohol oxidation.

The standard *β*-H elimination (Scheme 4, top) is the generally accepted mechanism for the alcohol oxidation reaction [37,38]. Starting from a complex (**A-B**), it involves an intramolecular Pd-assisted deprotonation, that leads to **C**, followed by a water elimination to **Cβ** and a *β*-hydride elimination that leads to **Dβ**. It has been confirmed by a significant number of theoretical studies [39,40,41,42,43].

In 2006, Goddard et al. proposed a “reductive *β*-elimination” for an aerobic alcohol oxidation catalyzed by Pd-NHCs’. It was shown to be slightly more favorable (by about 3 kJ/mol) compared to the classical *β*-hydride elimination [44,45], but it does not apply to our case of an anaerobic alcohol oxidation (an enone reoxidant is in excess in the reaction media to complete the catalytic cycle). Hence, we discarded Goddard’s pathway.

Another interesting pathway would involve an outer sphere-mechanism involving the participation of the P-O-H-O-P moiety, as described by van Leeuwen et al. [46]. However, through a study of the isomerization of *cis*-stilbene, we provided strong indications that a metal hydride intermediate was involved in our case (see ESI pp 9–10). We showed previously that an HO^−^ X-type ligand at the metal center was required for a positive outcome of the process [31]. Moreover, the absence of BrØnsted base effect on the reaction rate (ESI 2.7 p14) and the non-detection of a Pd alcoholate by ESI-MS analysis of the Pd crude reaction mixture [30] led us to consider a pathway involving a direct H abstraction (Scheme 4, bottom). This second mechanism has been considered for instance by Sheldon et al. [47] with Pd-OH as an effective catalytic species. It proceeds through a hydrogen bond-assisted reorganized system **(A-B)’** and a direct concerted six-membered ring hydride abstraction that leads to **D’**. This direct mechanistic pathway is rarely considered [38,47] We computationally investigate it and compare it to the standard *β*-H elimination route.

#### 2.2.1. *β*-Hydride Elimination vs. Direct H Abstraction

**Scheme 5:**
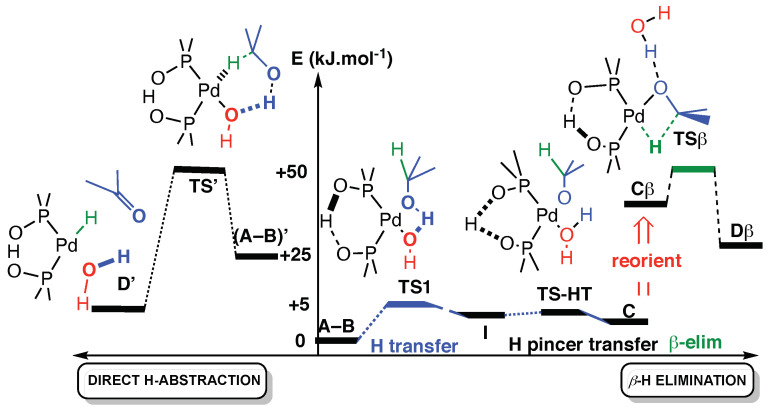
Comparison of the two calculated paths, plotted as a function of arbitrary reaction coordinates. To the right: proton transfer and β-H elimination. To the left direct mechanism. For the β-H elimination, a reorganization around metal is necessary to free a position (C → Cβ) (see the text and Table 2 for the values of the energies).

The *β*-H elimination, as shown in Scheme 5, requires that the alcohol’s proton first transfers to the hydroxyl ligand (**TS1**), then the proton of the pincer transfers within the PAP pincer from one oxygen to the other (**TS-HT**). It shall be noted that these proton transfers (**A-B** → **TS1** → **I** → **TS-HT** → **C**) have very low barriers on a shallow surface (see Table 2).

**Figure 2:**
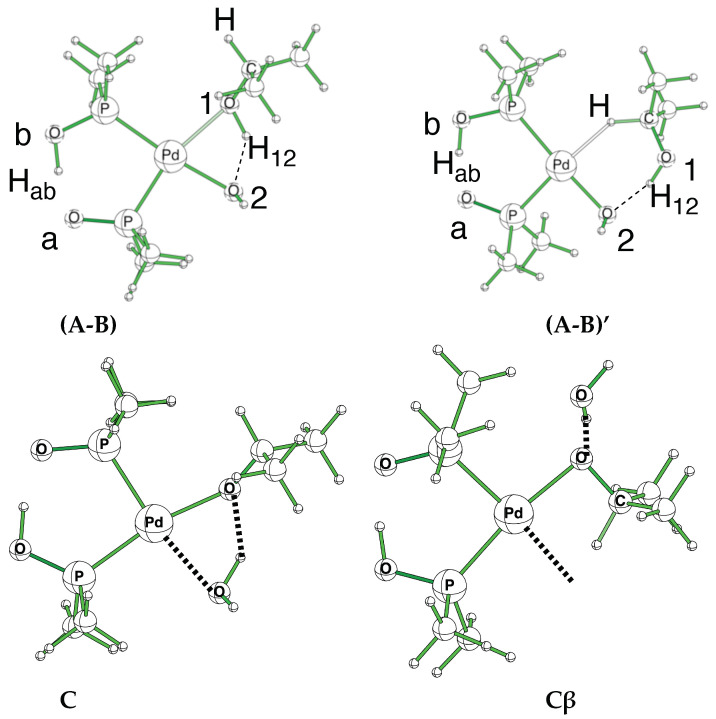
**(A-B)** and **(A-B)’** conformations and labels used to differentiate the key oxygen and hydrogen atoms. The proton in the pincer is labeled H_ab_, while the proton of the alcohol is labeled H_12_. Structures **C** and **Cβ** show the reorientation in the catalyst to free a position during the *β*-elimination.

For the last step of *β*-elimination (**Cβ** → **TSβ** → **Dβ**), we need to free a position in the square planar metal complex. We considered that this water molecule could either be removed, as stated previously, or stay in interaction in the complex with a rotation that frees a position on the square planar catalyst (reorientation). If water is removed, it will bind to another (external) molecule nearby, alcohol or water, and they will bind together by about 13.5 kJ·mol^−1^ [48]. Even if this was taken into account, the removal of the water molecule was found about 20 kJ·mol^−1^ higher in energy than the reorientation. With this in mind, we kept the water molecule during the *β*-elimination path and simply reoriented the groups of atoms (**C** → **Cβ** in Figure 2). For the Pd/PAP catalyst, the *β*-elimination mechanism has its highest point of the path at 55.6 kJ·mol^−1^ above (**A-B**). Similar calculations for the Pt/PAP catalyst gave similar energetics (55.9 kJ·mol^−1^—Table 2).

The direct H-abstraction mechanism is a one-step mechanism that requires an initial rearrangement of the reactant from **(A-B)** to **(A-B)’**. In **(A-B)’**, the alcohol molecule is reoriented in such a way (Figure 2) that the metal hydride is made in the same step as H_2_O. That **(A-B)’** conformation is 24.8 kJ·mol^−1^ over **(A-B)**, and the transition state **TS’** that leads to the **D’** product is at 55.9 kJ·mol^−1^, which is very close in energy to **TSβ** (55.6 kJ·mol^−1^).

These computations were also carried out for the Pt-PAP catalyst. The energies were similar, although a slightly lower path was obtained for the direct oxidation with Pt and **TS’** being at 51.3 kJ·mol^−1^.

We can see from this study that the direct H-abstraction mechanism is comparable in energy to the two-step *β*-hydride elimination, and it shall be relevant is some cases, for instance for the Pt analog.

We also note that the energetic balance of the oxidation can be evaluated by the energy difference between the separated reactants (Cat-OH + Alcohol) and separated products (Cat-H + H_2_O + ketone). It does not depend on the mechanism, and is unfavorable (by about 40 kJ·mol^−1^). This indicates that the choice of the sacrificial enone reoxidant is crucial for the catalytic cycle to proceed.

In the next section, we wish to better describe the behavior of this system on the path, around the transition state. For the sake of simplicity, only the direct mechanism is described.

#### 2.2.2. Reaction Force Analysis

**Figure 3:**
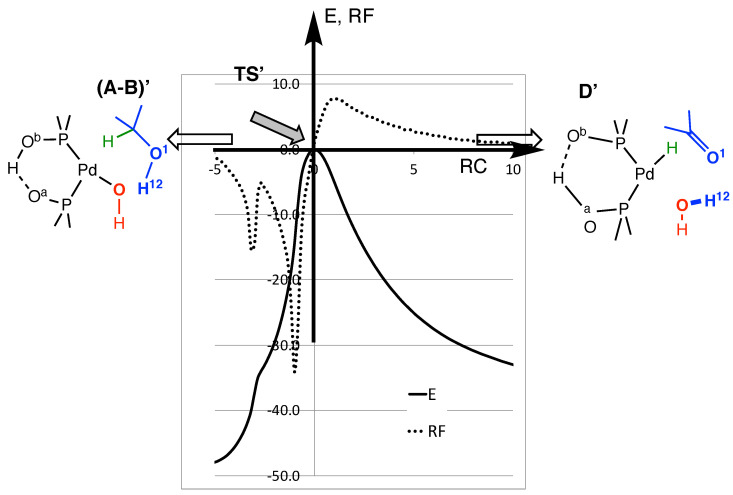
Energy (plain) and reaction force (dots) plots for direct H abstraction (arbitrary units) as a function of the reaction coordinate (RC). The energy is not ZPC-corrected. The energy of TS’ is set to zero. The RC axis corresponds to 125 points along the path, and the unit (RC_unit_) is arbitrary. The origin corresponds to geometry of the transition state **TS’**, and this RC axis is oriented in such a way that **(A-B)’** is on the left, and **D’** on the right.

The energy (E) and reaction force (RF) plots of the direct mechanism are displayed in Figure 3. The reaction processes from the left **(A-B)’**, alcohol reactive) to the right (**D’**, ketone formation), with a focus on the transition state **TS’** region. **TS’** is perfectly located at RC = 0, but both **(A-B)’** and **D’** would be outside the figure, along the horizontal axis. The plots are along the IRC path. They follow the reaction coordinate (RC). The IRC path converges very slowly to **D’**, and because the plots are close to the transition state, it is not possible to reach the **D’** geometry. The same applies to **(A-B)’**, and the global exothermicity of the reaction cannot be read from the energy curve. This would require a further reorganization of the reactive on the left, and of the product on the right. The energy does not include the ZPC; thus, the estimated barrier height appears higher than the value reported in Table 2.

**Figure 4:**
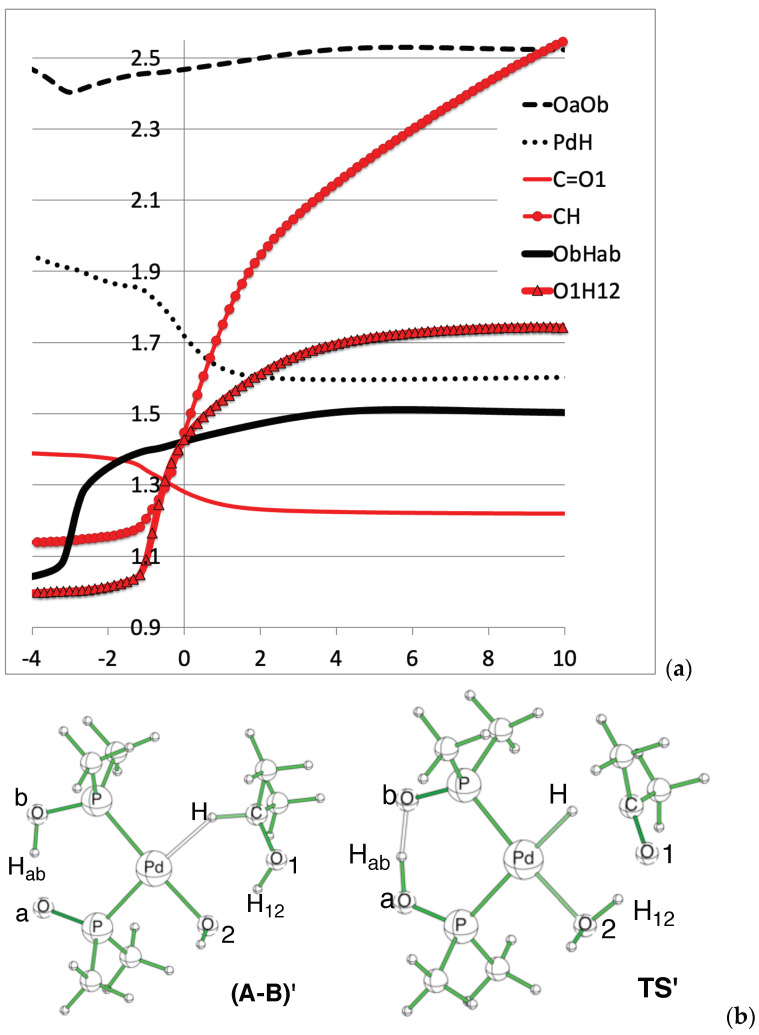
Direct H-abstraction variation of selected distances (in Å). (**a**) Distance variation plots. (**b**) Geometry and atom numbering.

The dotted curve shows the variation in the reaction force (RF) (Equation (1)) along RC (in kJ/mol/RC_unit_). It is set that RF = 0 at the geometry of **TS’**, and as usual [49,50,51]. It is negative on the left of the transition state, and positive on the right. RF shows an unusual shape, with two extrema before the transition state, instead of one. Each extremum corresponds to a chemical event during the reaction [52,53]. To better illustrate those two events, we plotted the distance variation during the IRC path (Figure 4). The RC axis is the same as that of Figure 3.

The reaction corresponds to the alcohol oxidation, and the C=O1 double bond is formed. Consistently, it can be seen on Figure 4 that the C-O1 distance decreases from about 1.4Å to about 1.2Å. Simultaneously, the CH and O_1_H_12_ distances increase (red curves). This corresponds to the direct oxidation mechanism, where both hydrogens migrate from the alcohol. This event corresponds to the second peak, at RC = −1, of the RF curve (Figure 3). The first peak in RF, at RC = −3, corresponds to the H_ab_ transfer in the PAP pincer, from O_b_ to O_a_. The black curve (Figure 4) shows that the O_b_-H_ab_ distance varies from about 1.1Å to 1.5Å. The dash curve in the top part of Figure 4 shows that the O_a_O_b_ distance slightly shortens to ease the transfer [54].

The CH distance is not converged, but rather rises continuously, which is consistent with the aforementioned reorganization around the Pd atom, which is not completed during the IRC.

**Figure 5:**
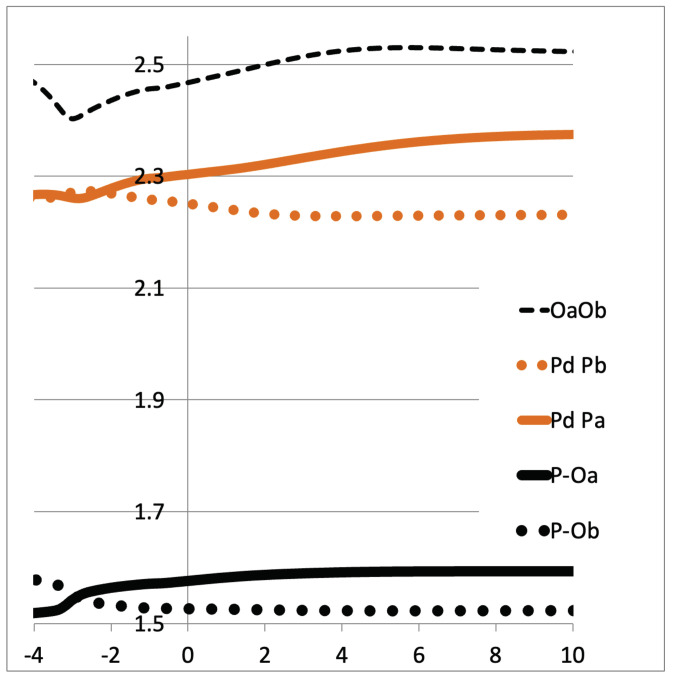
Direct H abstraction: variation of Pd-P’s distances (in Å). P_b_ corresponds to the P atom that bears O_b_.

During the H_ab_ transfer another reorganization takes place in the catalyst. Here, we have the opportunity to better describe the equilibrium between phosphinito–phosphinous acid in the PAP ligand (Scheme 1b). It can be seen in Figure 5 that the Pd-P_a_ distance increases from ~2.25Å to ~2.35Å while Pd-P_b_ decreases. Meanwhile, the P-O’s distances vary similarly. It looks like the H_ab_’s transfer prepares the ligands so that P_a_ becomes a phosphinous acid ligand, in *trans* to the upcoming hydride. As such, we observe an elongated P_a_-O bond and a larger Pd-P_a_ distance (2.35Å), more like an L-type ligand. On the contrary, P_b_ becomes more like a phosphine oxide (phosphinito) ligand, close to an X-type ligand (Pd-P_b_ = 2.15Å), in *trans* to the upcoming water ligand. It is shown that the PAP ligand continuously adapts its electronic structure during the reaction path by the pincer’s proton transfer.

## 3. Materials and Methods: Computational Details

All calculations were performed at the B3LYP/def2TZVP level with Grimme’s dispersion terms D3 [55,56,57,58]. The transition states were checked with analytic second derivative analysis. They always have a unique imaginary frequency.

For a deeper understanding of PAP ligand’s behavior, we performed a reaction force analysis within the Intrinsic Reaction Coordinate (IRC) model [59]. An analytical calculation of the second derivatives was requested at each of the 125 steps of the IRC, and the path energies were used to obtain the reaction force through a numerical derivative of the energy with respect to the reaction coordinate (1) [50].

(1) $$RF\left(RC\right)=-\frac{\Delta E(RC)}{\Delta RC}$$

The calculations were carried out with the Gaussian 09 software [60] with the default parameters, notably for the B3LYP method. Except for the IRC’s energies, the Zero Point Correction (ZPC) is included.

## 4. Conclusions

The self-assembled PAP ligand presents a very specific behavior during this catalytic alcohol oxidation. The hydrogen bond assisted linkage (PAP pincer) makes it unique and significantly different to purely covalent bidentate diphosphines. This study demonstrated that the Pd/PAP and Pt/PAP performances for anaerobic alcohol oxidation could not be matched by conventional commercial phosphorus ligands. We also highlighted an unprecedented direct H-abstraction mechanism instead of classical β-hydride elimination for the same reaction. Mentioned in 2002 by Sheldon et al. [47], to the best of our knowledge, this concerted hydrogen bond-assisted pathway has not been studied by molecular modeling. The analysis of reaction forces revealed a continuous adaptive modulation of the PAP ligand electronic properties, and led us to give a plausible explanation for this unusual mechanism. This advance in PAP ligand behavior understanding would probably give further help to visit challenging reactions in catalysis. It demonstrated that M/PAP catalysts feature an unusual operating mode, allowing us to dismantle the preconceived idea that metal-catalyzed reactions necessarily involve the formation of a metal alcoholate and then a *β*-H elimination.

## Figures and Tables

**Table 1:**
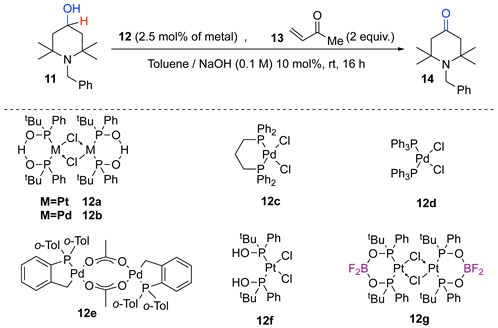
Comparison between M/PAP complex **10** and structurally similar catalysts ^a^.

**Entry**	**Catalyst**	**Yield (%) ^b^**	**Reaction Mixture Aspect**
1	**12a**	91	Translucent
2	**12b**	36	Translucent
3	**12c**	0	Black Pd deposit
4	**12d**	10	Black Pd deposit
5	**12e**	0	Black Pd deposit
6	**12f**	8	Black Pt deposit
7	**12g**	74	Translucent

^a^ Reaction conditions: **11** (0.6 mmol), **12** (0.025 mmol of metal), **13** (1.2 mmol), toluene (6 mL), NaOH (0.1 M, 0.6 mL), 20 °C; ^b^ Isolated Yield for **14** after purification by column chromatography.

**Table 2:** B3LYP/def2TZVP relative energies (kJ·mol^−1^) with D3 dispersion and including Zero Point Correction (ZPC) ^a,b^.

	Pd-PAP	Pt-PAP
Separated reactants	82.9	84.2
*β*-H Elimination		
(A-B)	0.0	0.0
TS1	4.3	6.5
I	4.7 ^a^	- ^b^
TS_HT	−0.5 ^a^	- ^b^
C	3.0 ^a^	4.2
(the substrate and H_2_O are reoriented)		
C_β_	29.8	38.4
TS_β_	55.6	55.9
D_β_	44.9	21.8
Direct H abstraction		
(A-B)’	24.8	25.0
TS’	55.9	51.3
D’	13.4	9.7
Separated products	122.7	125.5
Energetic Balance	39.7	41.3

^a^ When the ZPC is taken into account, some distortion of the potential energy surface can push an intermediate over a transition state. ^b^ The intermediate **I** could not be located for Pt-PAP, so the proton transfer in the pincer is only a shoulder on the path.

## Data Availability

The original contributions presented in the study are included in the article/Supplementary Material, further inquiries can be directed to the corresponding author/s.

## Supplementary Materials

The following supporting information can be downloaded at: https://www.mdpi.com/article/10.3390/molecules29214999/s1. References [61,62] are cited in the Supplementary Materials.
