# Supplementary material for: The Adaptative Modulation of the Phosphinito–Phosphinous Acid Ligand: Computational Illustration Through Palladium-Catalyzed Alcohol Oxidation
Source: Molecules. 2024 Oct 22;29(21):4999. doi: 10.3390/molecules29214999 (PMC11547856; doi:10.3390/molecules29214999)
Supplement: Supplementary file 1 [file molecules-29-04999-s001.zip › molecules-3243168-supplementary.pdf]

The Supporting Information

for

Adaptative modulation of the phosphinito-phosphinous acid ligand: computational illustration through palladium catalyzed alcohol oxidation

Romain Membrat,[1] Etonam Tété Kondo,[1] Alexis Agostini,[1] Alexandre Vasseur,[1,2] Paola Nava,[1] Laurent Giordano,[1] Alexandre Martinez,[1] Didier Nuel,*[1] Stéphane Humbel*[1]

[1] Aix Marseille Univ, CNRS, Centrale Med, ISM2, Marseille, France [2] Université de Lorraine, CNRS, L2CM, F-54000 Nancy, France

* Correspondence: DN: didier.nuel@centrale-med.fr,, SH: stephane.humbel@univ-amu.fr

Sommaire

1. General information ... 4

1.1 Solvents ... 4

1.2 Thin layer chromatography ... 4

1.3 Nuclear Magnetic Resonance ... 4

1.4 Physical and Analytical Measurements ... 4

2. Experimental part ... 5

2.1 Catalysts synthesis ... 5

2.2 Palladium and platinum catalyzed alcohols oxidation system ... 5

2.3 Dissymmetric nature of the PAP ligand in Pt/PAP complex at the solid state ... 7

2.4 Isomerization of *cis*-stylbene ... 9

2.5 Non detection of a palladium alcoholate by ESI-MS crude mixture analysis ... 11

2.6 Kinectic studies ... 13

2.7 Brønsted base effect study ... 15

2.8 NMR spectra ... 16

3. Computational work ... 17

3.1 Main Structures ... 17

3.1.1 TS1_min1 ; label = (A-B) ... 17

3.1.2 TS1 ; label = TS1 ... 18

3.1.3 TS1_TP_min1 ; label = I ... 21

3.1.4 TS1_TP ; label = TS_HT ... 22

3.1.5 TS1_min2 ; label = C ... 23

3.1.6 TS2_min1 ; label = Cβ ... 24

3.1.7 TS2_H2O ; label = TSβ ... 26

3.1.8 TS2_min2 ; label = Dβ ... 27

3.1.9 TS1-2_min1 ; label = (A-B)' ... 30

3.1.10 TS1-2 ; label = TS' ... 31

3.1.11 TS1-2_min2 ; label = D' ... 34

3.2 Additionnal Structures ... 36

3.2.1 H2O ... 36

3.2.2 cetone ... 36

3.2.3 propanol ... 36

3.3 removing water from the ß elimination path 36
3.3.1 TS2_min1 ; label = Cβ 37
3.3.2 TS2 ; label = TSβ 38
3.3.3 TS2_min2 ; label = Dβ 39
4. References 40

1. General information

1.1 Solvents

All solvents were purified by standard procedures or obtained from a Solvent Purification System (Braun SPS 800).

1.2 Thin layer chromatography

Thin layer chromatography (TLC) was carried out on Merck silica gel 60 F_{254} and visualized under ultraviolet light (254nm and 366nm), and/or through spray with a basic $KMnO_4$ solution followed by heating with a heat gun. Flash chromatography was performed on Merck silica gel 60 (230-400Mesh) unless otherwise noticed.

1.3 Nuclear Magnetic Resonance

^{1}H, ^{13}C and ^{31}P spectra were recorded on Bruker Avance III nanobay spectrometers operating at 400 and/or 300MHz.^{13}C and ^{31}P nuclei were observed with proton decoupling. Unless otherwise specified NMR spectra have been recorded using $CDCl_3$ as solvent. 85% phosphoric acid was used as external reference for ^{31}P spectra,. Chemical shift (δ) of ^{1}H and ^{13}C are reported in ppm relative to TMS (based on the signal of residual $CHCl_3$ in $CDCl_3$ at δ=7.27 ppm). Coupling constants (J) values are given in Hz. Proton NMR information is given in the following format: mutiplicity (s, singulet; d, doublet; t, triplet; at, apparent triplet; q, qartet; m, multiplet), coupling constant J, integration. The prefix br indicates that the signal is broadened.

1.4 Physical and Analytical Measurements

High Resolution MS experiments were performed with a SYNAPT G2 HDMS (Waters) equipped with an electrospray ionization (ESI) source. In the positive ion mode, the capillary voltage was set at 2800V and the cone voltage was set between 10-55V. In this hybrid instrument, ions were measured using an orthogonal acceleration time of flight (oa-TOF) mass analyzer. In MS, accurate mass measurements were performed using two reference ions from polyethylene glycol or polypropylene glycol internal standards.

2. Experimental part

2.1 Catalysts synthesis

Catalysts **12a**, **12b** and **12f** and corresponding ligands were prepared according to our reported procedures.[1–3] For BF_2 insertion, Leung procedure was used on platinum complexes (not optimized).[4]

Procedure for 12g synthesis

In a flame dried 50 mL Schlenk tube was prepared a solution of 30 mg of the starting complex **12a** in 15 mL of dry Et_2O under argon atmosphere. The solution was cooled down to 0 °C and 15 equivalents of $HBF_4{\cdot}Et_2O$ solution were dropwized carefully. After a 30 minutes stirring period at room temperature, the mixture was concentrated under vacuum and diluted in water and Et_2O. After removing the aqueous layer, the organic layer was dried over $MgSO_4$ and concentrated under vacuum. The residue was diluted in hexane / acetone / Et_2O and concentrated several times until obtention of a well defined white powder.

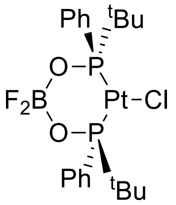

Compound 12g (642 g/mol) was obtained according to the general procedure from compound **12d** as a white powder in 88 % isolated yield. **^{1}H NMR (300 MHz, $CDCl_2$)** *δ*, 7.92 (m, 2H), 7.48 (m, 3H), 1.11 (d, J = 16.1 H, 9H) **^{31}P NMR (162 MHz, CD_2Cl_2)** : *δ* 77 (s, 0.7 P), 77 (d, J = ~ 4000 Hz , 0.3 P); **^{19}F (282 MHz, CD_2Cl_2)** δ - 138

2.2 Palladium and platinum catalyzed alcohols oxidation system

The model substrate **11** used for the experimental study was prepared according to our previously published methodology using microwave assisted heating.[5]

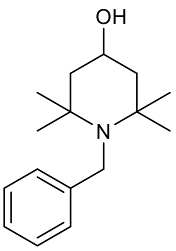

***N*-benzyl-2,2,6,6-tetramethylpiperidin-4-ol** (**11**, MW=247.4 g/mol). **^{1}H NMR 400MHz $CDCl_3$** : δ 7.43 (m, 2H), 7.28 (m, 2H), 7.15 (m, 1H), 4.05 (m, 1H), 3.83 (s, 2H), 1.91 (dd, J=4.07 ; 12.10 Hz, 2H), 1.5 (t, J=11.63Hz, 2H), 1.26 (s, 1H), 1.21 (s, 6H), 0.95 (s, 6H) ; **^{13}C NMR 101MHz $CDCl_3$101 MHz $CDCl_3$:** δ 145.3, 127.8, 126.6, 125.6, 64, 56.2, 50.3, 47.2, 33.8, 22.5; **IR (ATR)** 3249, 2980, 2931, 1460, 1449, 1379, 1368, 1256, 1187, 1174, 1162, 1040, 1025, 955, 938, 844, 722, 605 cm^{-1};

Oxidation at 105 °C in neutral conditions[6]

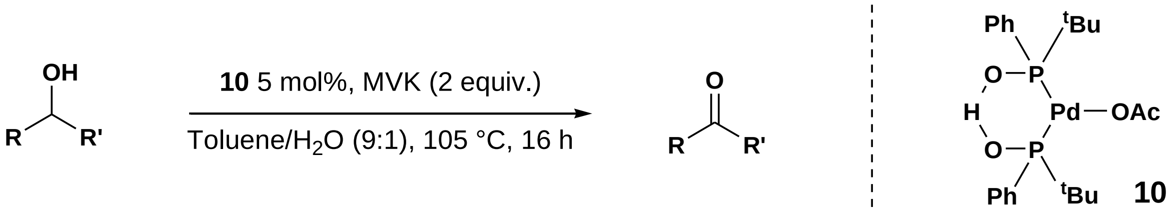

A 10-mL Schlenk tube, equipped with a stir bar and rubber septum was charged with catalyst **10** (0.025 mmol), toluene (8 mL), distilled water (1 mL), alcohol (1 mmol), methyl vinyl ketone (463 µL, 5 mmol) and placed under an inert atmosphere. The reaction mixture was then stirred at 105 °C (oil bath) for 16 h. After cooling down the mixture to rt, the layers were separated and the aqueous phase was extracted with Et_2O (twice). The combined organic fractions were washed with brine, dried over Na_2SO_4, filtered and concentrated in vacuum. The products were obtained after purification by silica-gel chromatography (SiO_2, petroleum ether/Et_2O, 90:10-50:50 or CH_2Cl_2/MeOH, 95:5-90:10).

Oxidation at room temperature in weakly basic conditions[2]

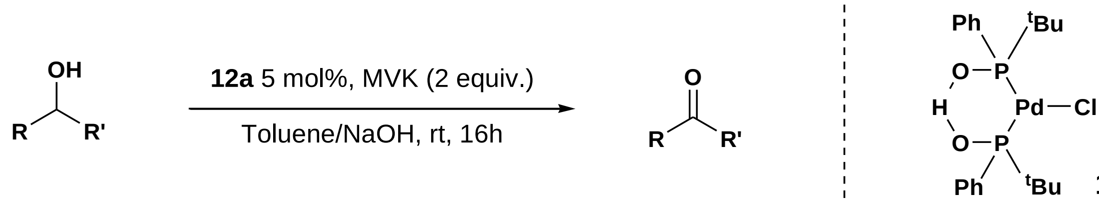

12a

A flame-dried Schlenk tube was charged under argon atmosphere with the catalyst **12** (0.025 equiv.) and the alcohol (1 equiv.) and the dissolved in dry toluene (6 mL). Methyl vinyl ketone (2 equiv.) and aqueous NaOH 0.1M (0.1 equiv.) were then added to the reaction mixture. After overnight stirring, the reaction was stopped (or warmed up to 50°C until complete conversion of the alcohol). Layers were separated and the aqueous layer was extracted 3 times with 5 mL of ethyl acetate. Combined organic layers were dried over Na_2SO_4 and concentrated under vacuum. The resulting crude product could be purified if necessary by column chromatography on silica gel using an appropriated combination of petroleum ether and ethyl acetate to yield pure desired products.

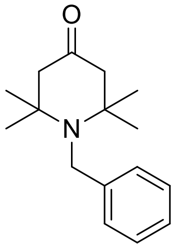

***N*-benzyl-2,2,6,6-tetramethylpiperidin-4-one 14** (MW=245.4 g/mol) was obtained according to the general procedure from compound **11** as a white solid (94%) after purification by flash chromatography[1]. **^{1}H NMR 400 MHz $CDCl_3$** : δ 7.5 (m, 2H), 7.30 (m, 1H), 7.20 (m, 2H), 3.93 (s, 2H), 2.48 (s, 4H), 1.14 (s, 12H) ; **^{13}C NMR 101 MHz $CDCl_3$** : δ 209.9, 144.6, 128.0, 126.6, 125.9, 60.1, 55.9, 47.6

2.3 Dissymmetric nature of the PAP ligand in Pt/PAP complex at the solid state

di-*μ*-hydroxotetrakys-[(*R_P*)-tert-butylphenylphosphinito-*κ*-*P*]diplatinate(2-).[2]**CCDC 1582905**:
Recrystallised by slow evoparation in hexande / DCM. Most of hydrogens are omitted for clarity

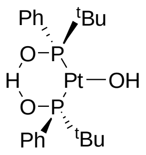

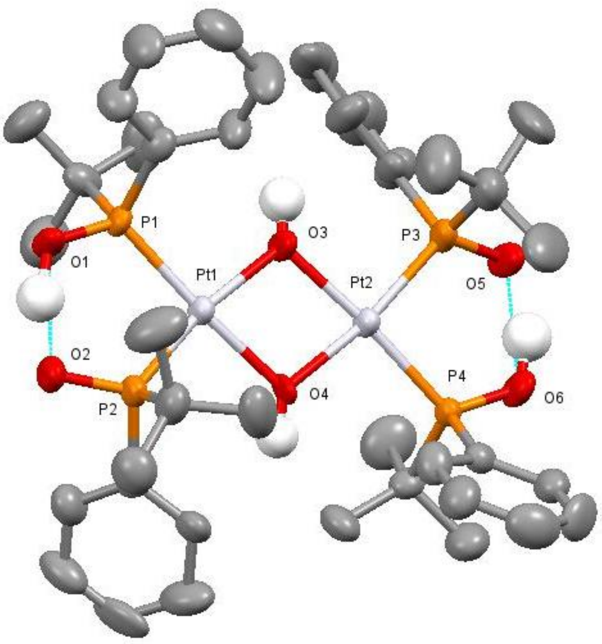

**Pt(1)-P(1)**	**2.232(3)**	O(4)-Pt(1)-P(1)	174.6(3)
**Pt(1)-P(2**	**2.227(4)**	O(4)-Pt(1)-P(2)	93.9(3)
Pt(1)-O(3	2.146(10)	O(4)-Pt(1)-O(3)	76.8(4)
Pt(1)-O(4)	2.131(9)	P(3)-Pt(2)-P(4)	91.59(14)
**Pt(2)-P(3)**	**2.234(4)**	O(3)-Pt(2)-P(3)	92.8(3)
**Pt(2)-P(4)**	**2.245(3)**	O(3)-Pt(2)-P(4)	175.1(3)
Pt(2)-O(3)	2.103(9)	O(3)-Pt(2)-O(4)	77.5(4)
Pt(2)-O(4)	2.140(9)	O(4)-Pt(2)-P(3)	168.9(3)
**P(1)-O(1)**	**1.553(10)**	O(4)-Pt(2)-P(4)	98.3(3)
**P(2)-O(2)**	**1.538(11)**	O(1)-P(1)-Pt(1)	116.6(4)
P(3)-O(5)	1.550(12)	O(2)-P(2)-Pt(1)	117.7(5)
P(4)-O(6)	1.524(10)	O(5)-P(3)-Pt(2)	117.5(5)
P(2)-Pt(1)-P(1)	91.43(14)	O(6)-P(4)-Pt(2)	116.9(4)
O(3)-Pt(1)-P(1)	97.9(3)	Pt(2)-O(3)-Pt(1)	103.2(5)
O(3)-Pt(1)-P(2)	169.7(3)	Pt(1)-O(4)-Pt(2)	102.4(4)

Other examples of X-Ray structure for Pd/PAP complexes are reported in our precedent publications.[2,3,7]

^{31}P NMR spectrum:

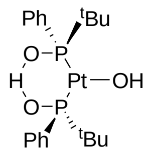

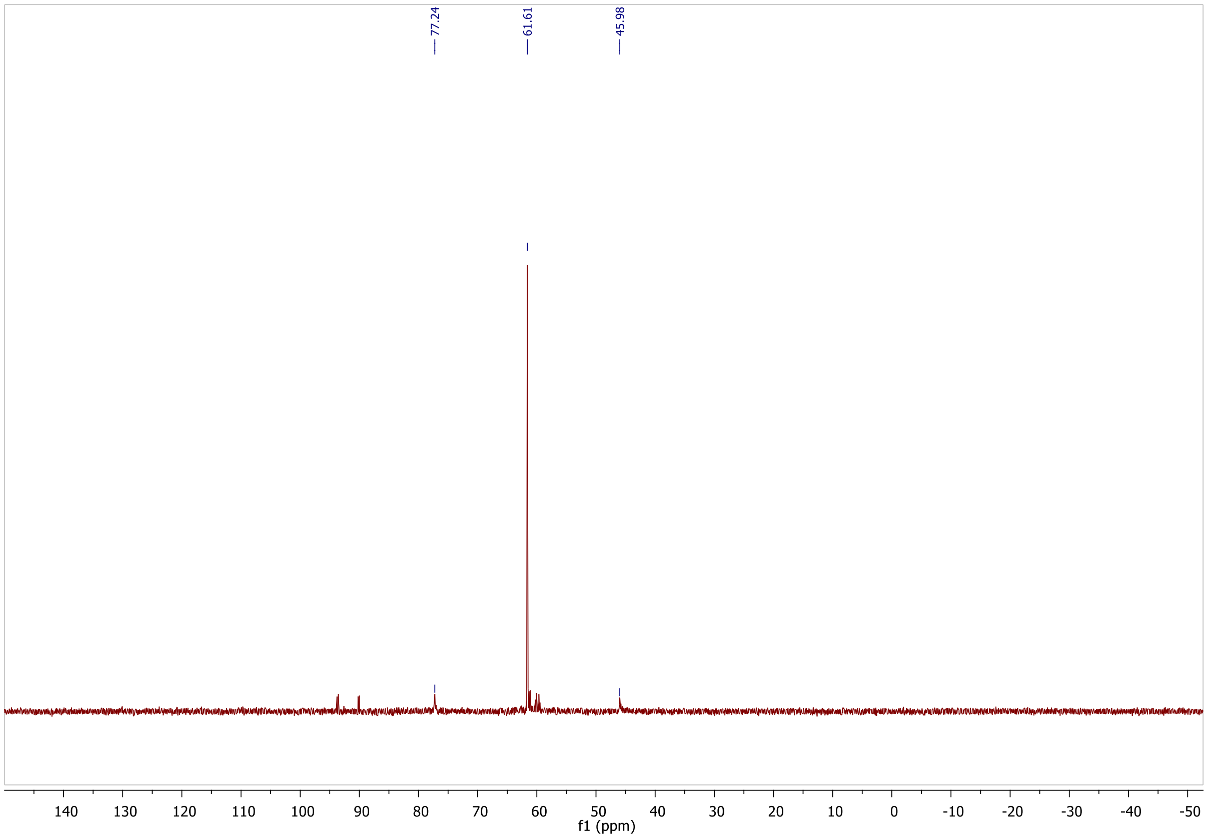

2.4 Isomerization of *cis*-stylbene

A 20 mL Schlenk tube was charged under argon atmosphere with the catalyst $[(^tBuPhPO)_2H)_2M(\mu\text{-}Cl)]_2$ (M=Pd, Pt)(0.025 mmol), *cis*-stylbene (1 mmol) and the hydride donor (1.5 mmol). Substrates were dissolved in toluene (8 mL) and water (1 mL, or NaOH 0.1M 0.6 mL). The reaction mixture was stirred overnight at room temperature for 16 h. Thereafter, the aqueous layer was removed, the organic layer was dried over Na_2SO_4 and concentrated under vacuum. *cis* / *trans* proportion was evaluated by integration of the olefinic proton in ^1H NMR.

***cis*-stylbène ^{1}H NMR (400 MHz, $CDCl_3$) :**δ 7.38-6.98 (m, 10H), 6.58 (s, 2H) ; **^{13}C NMR (400 MHz, $CDCl_3$):**δ 137.2, 130.2, 128.9, 128.2, 127

***trans*-stylbène ^{1}H NMR (400 MHz, $CDCl_3$) :**δ 7.48 (m, 4H), 7.34 (m, 4H), 7.21 (m, 2H), 7.15 (s, 2H) ;**^{13}C NMR (400 MHz, $CDCl_3$):** δ137.4, 128.7, 127.6, 126.5

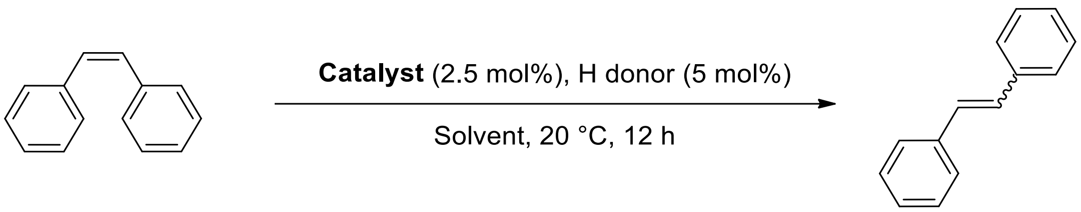

(*Z*) / (*E*) 100 : 0

Entry	H donor	Solvent	(*Z*) stylbene (%)[b]	(*E*) stylbene (%)[b]
**1**	-	Toluene, NaOH	100	0
**2**	1-phenyléthanol	Toluene, NaOH	0	100
**3**	Et_3SiH	Toluene, NaOH	0	100
**4**	Et_3SiH	Toluene	53	47
**5[a]**	Et_3SiH	Toluène, NaOH	100	0

Conditions : *cis*-stylbène (1 mmol), H donor (1.5 mmol), **Catalyst** (0.025 mmol), Toluene (10 mL), NaOH (0,1 M, 1 mL) 20 °C, 12 h ; [a] Without catalyst

Proposed mechanism for isomerization involving a M-H species

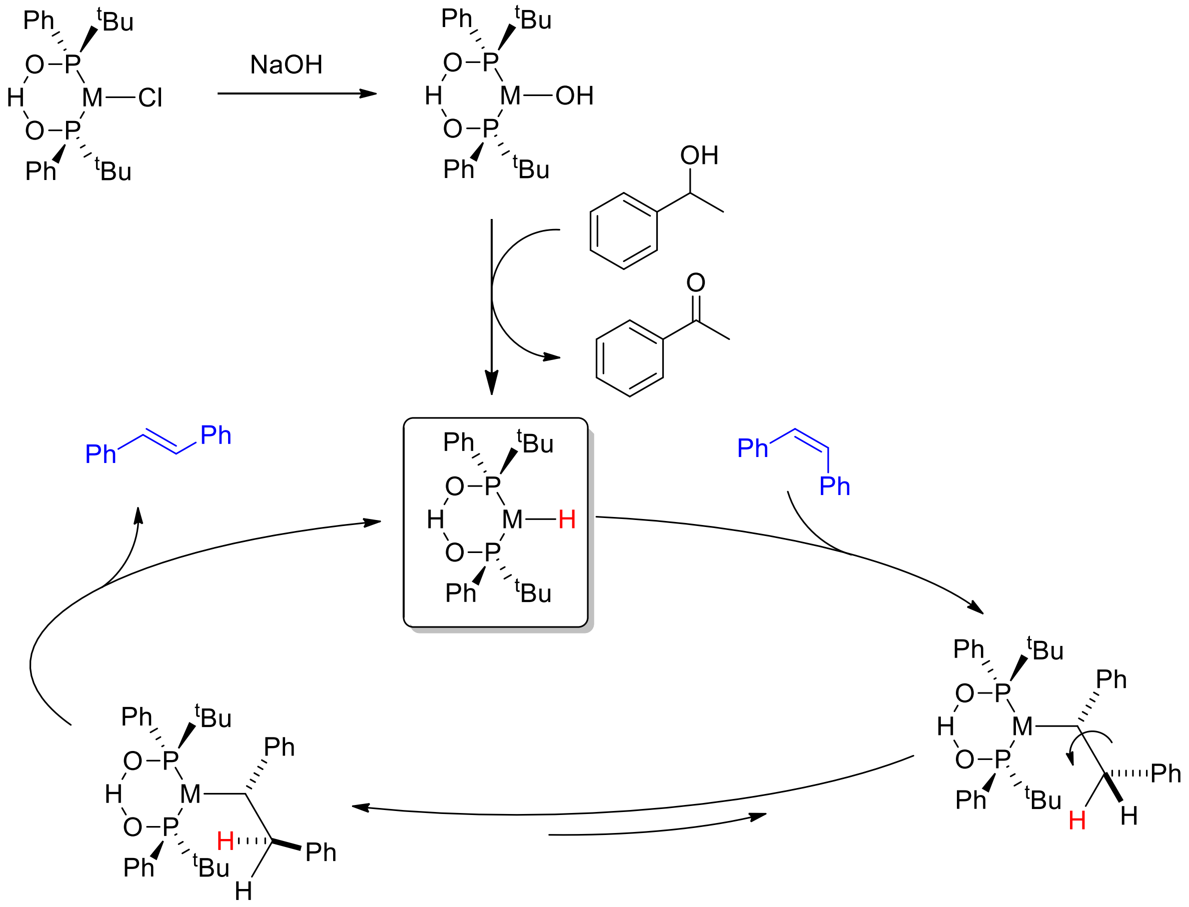

2.5 Non detection of a palladium alcoholate by ESI-MS crude mixture analysis

Procedure

A 10-mL Schlenk tube, equipped with a stir bar and rubber septum was charged with $[(^{t}BuPhPO)_2H)_2Pd(\mu\text{-}OAc)]_2$ (0.025 mmol), 1-phenylethanol (1 mmol), methyl vinyl ketone (1 mmol), toluene (8 mL), distilled water (1 mL) and placed under an inert atmosphere. The reaction mixture was then stirred at 105 °C (oil bath) for 15 min. The reaction mixture was then directly injected into the ESI-MS spectrometer (ESI-+-MS, + 20 V) after dilution in MeOH and analyzed at 105 °C.

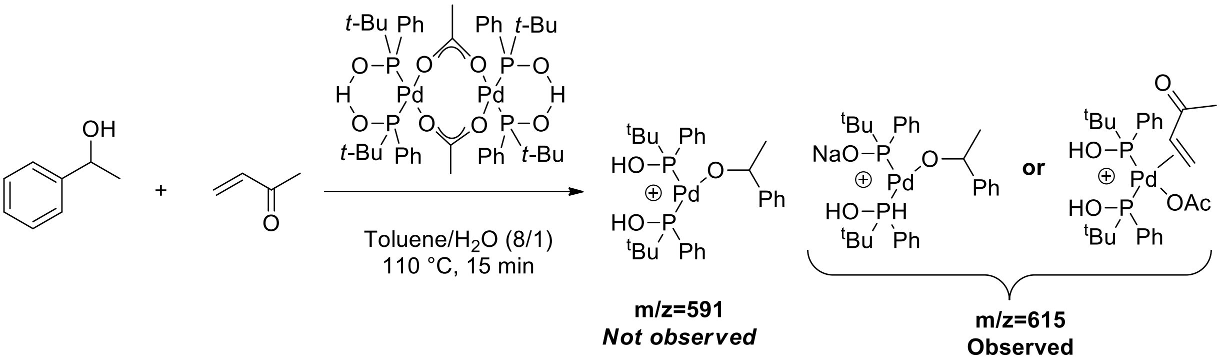

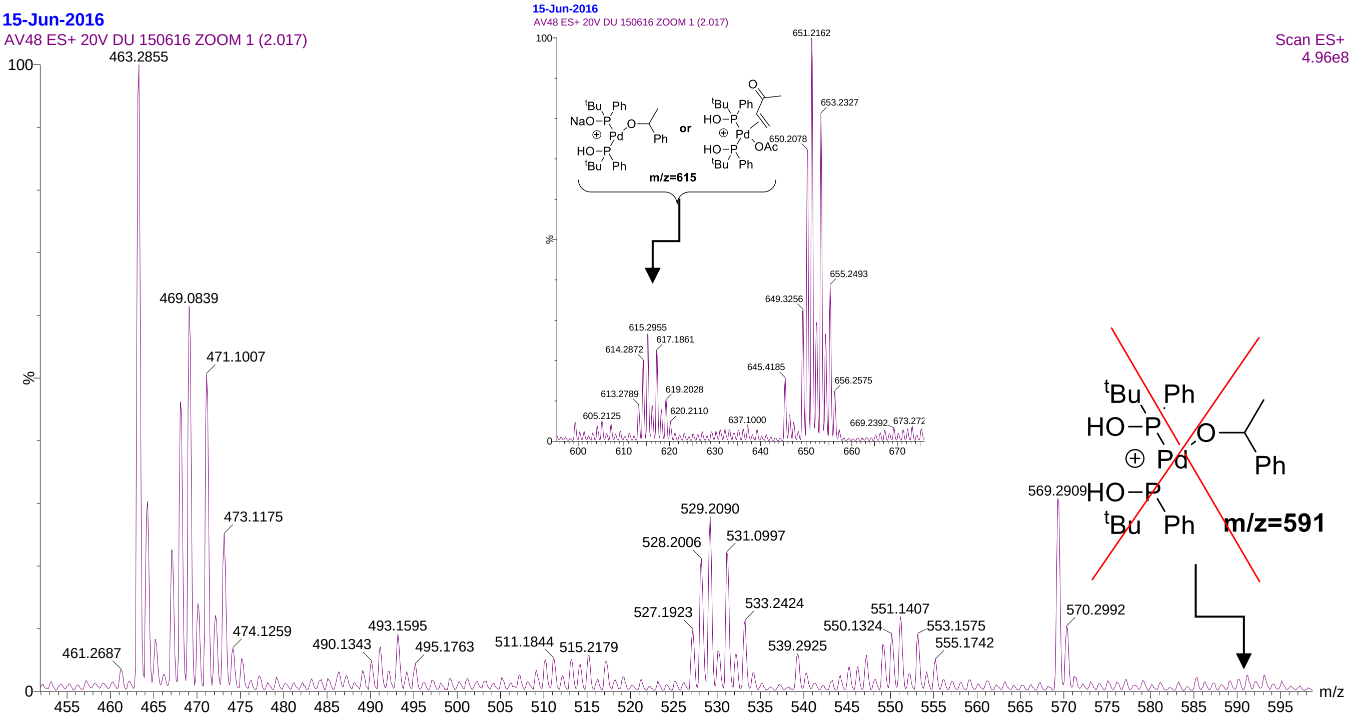

Two species could be affected to the m/z=615 massive. A complementary experiment with another alcohol was run to rule out the Pd-alcoholate.

Procedure

A 10-mL Schlenk tube, equipped with a stir bar and rubber septum was charged with (0.025 mmol), *p*-bromo-phenylethanol (1 mmol), methyl vinyl ketone (1 mmol), toluene (8 mL), distilled water (1 mL) and placed under an inert atmosphere. The reaction mixture was then stirred at 105 °C (oil bath) for 15 min. The reaction mixture was then directly injected into the ESI-MS spectrometer (ESI-(+)-MS, +20 V) after dilution in MeOH and analyzed at 105 °C.

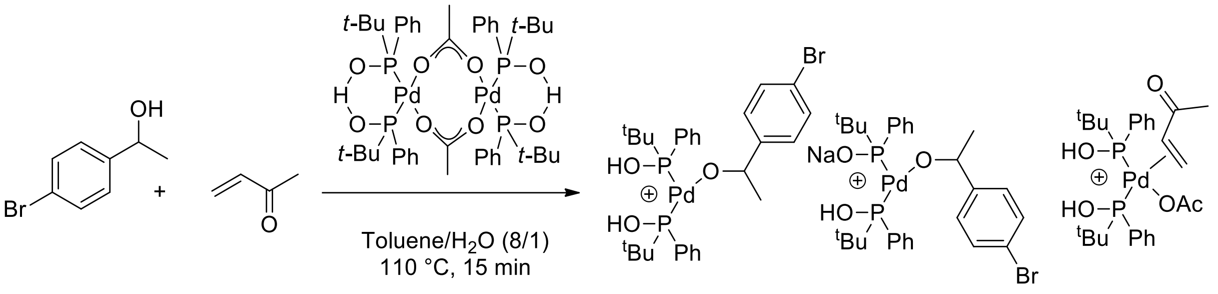

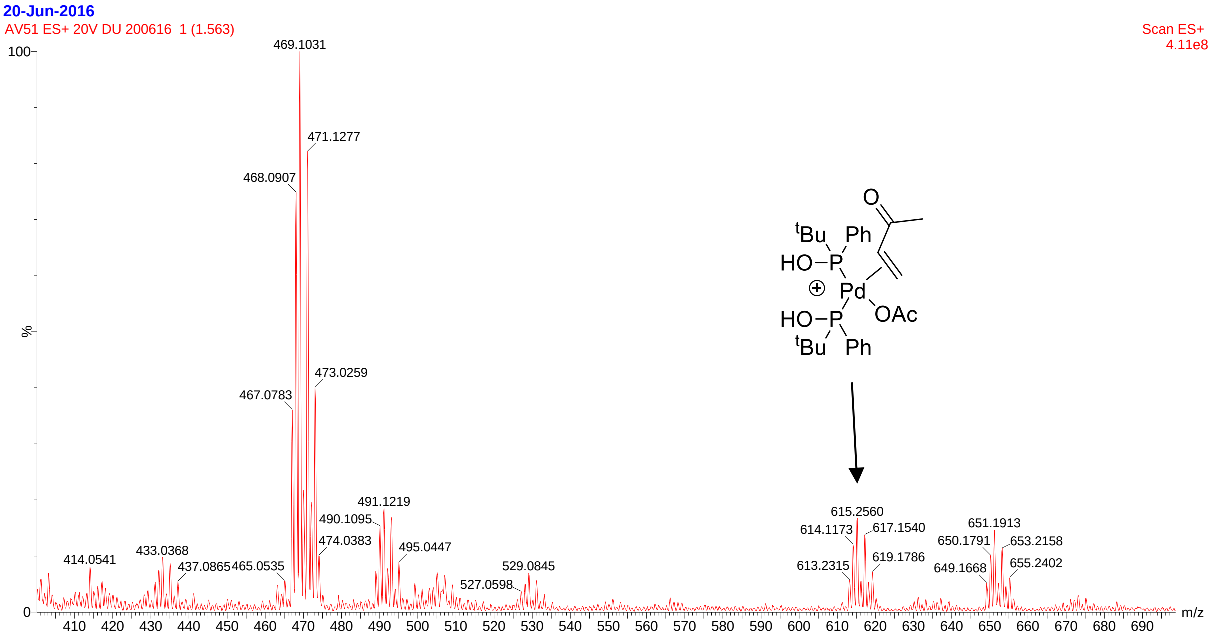

The massive at m/z=615 was still in the mixture when the experiment was conducted with *p*-bromo-phenylethanol. There is no shift of the signal toward m/z=669 or m/z=691. This signal at m/z=615 should be affected to the starting complex associated with the methyl vinyl ketone.

2.6 Kinetic studies

2.6.1 Initial kinetic rate and induction time

MVK = methyl Vinyl Ketone

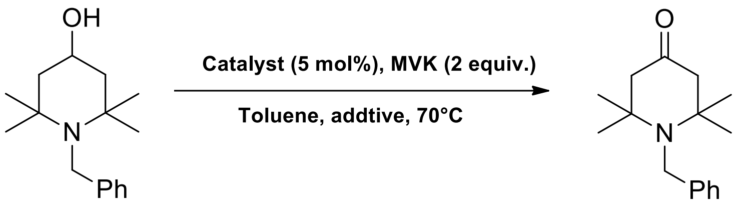

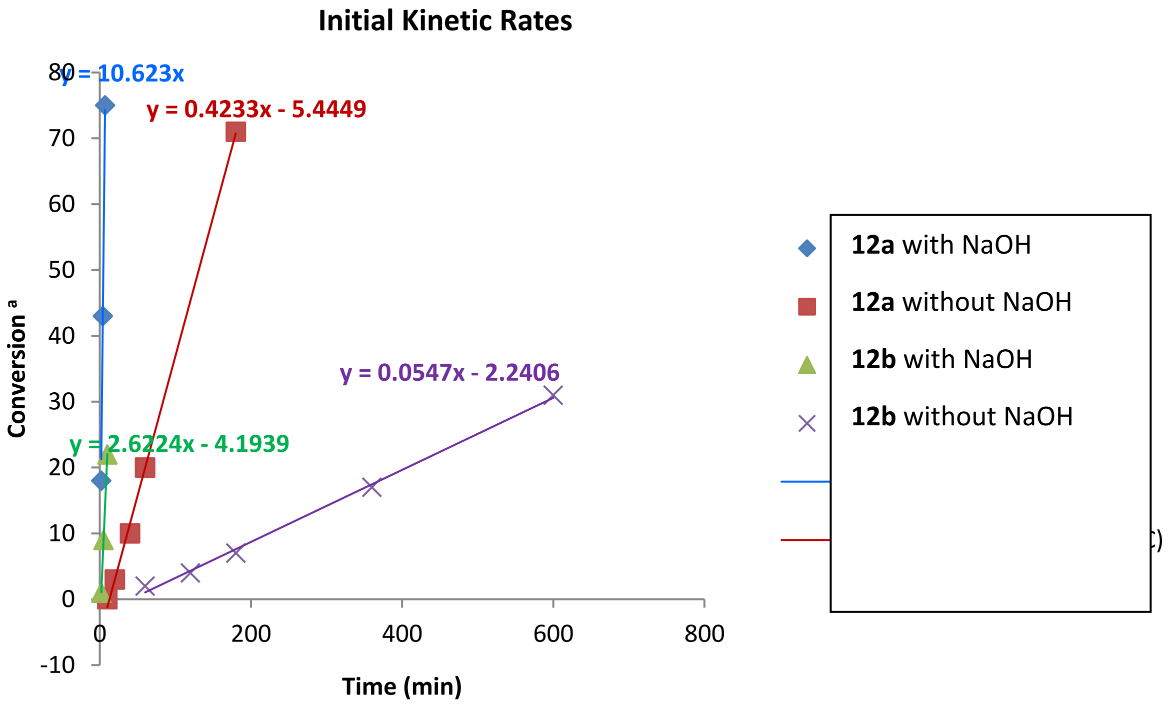

<u>Conditions :</u> Substrate **11** 0.4mmol (100mg), Catalyst **12** 5 mol% (0.02mmol), MVK 2 equiv. 1.2mmol, appropriated additive (NaOH 0.1M 10 mol% or H_2O 1mL) Toluene 8mL

[a] Conversions were measured by ^{1}H NMR. Profiles obtained by the arithmetic mean of 3 experiments.

2.6.2 First order determination

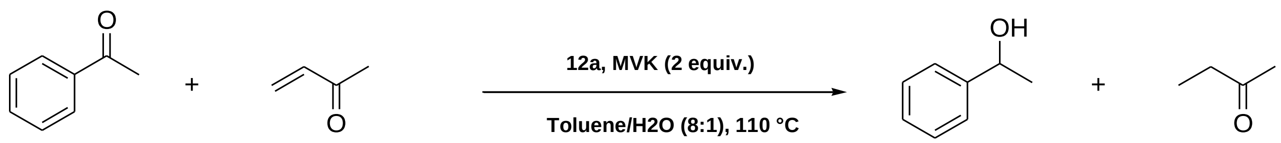

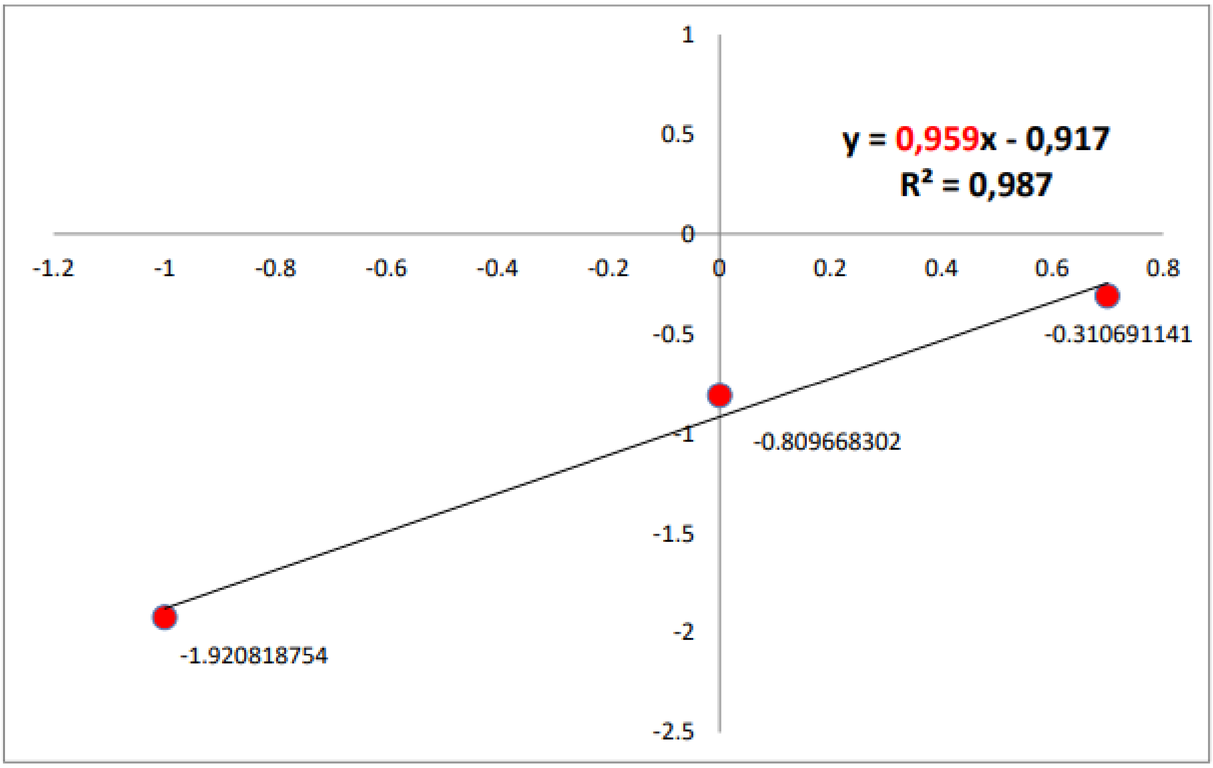

1-phenylethanol (1 mmol), MVK (1 mmol), **12a** (0.025, 0.005 and 0.0005 mmol in Pd), toluene (8 mL), H2O (1 mL) ; Yields determined by GC analysis with dodecane as internal standard

Order in Pd = 0.959 ≈ 1

Entry	Pd loading (mol %)	$k_{initial}$ (s^{-1})	Log($k_{initial}$)
**1**	5	0.489	-0,310691141
**2**	1	0.155	-0,809668302
**3**	0.1	0.012	-1,920818754

2.7 Brønsted base effect study

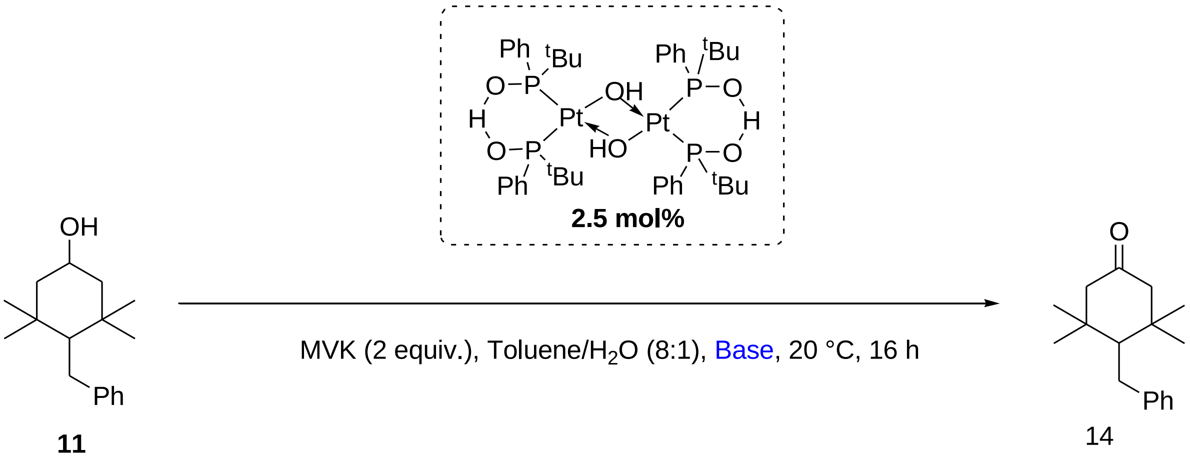

pH constant

Base =	
**NaOH**	**86%**
**K_2CO_3**	**12%**
**K_3PO_4**	**15%**
**Na_2CO_3**	**10%**

To highlight a Brønsted base effect due to the use of NaOH additive, we try to add to the reaction mixture small amounts of Brønsted inorganic base solution to reach the same pH. It should be stressed that the good conversion obtained with NaOH is not reproduced by using an inorganic basic solution at the same pH. It demonstrated that the effect of NaOH is not only a Brønsted base effect.

2.8 NMR spectra

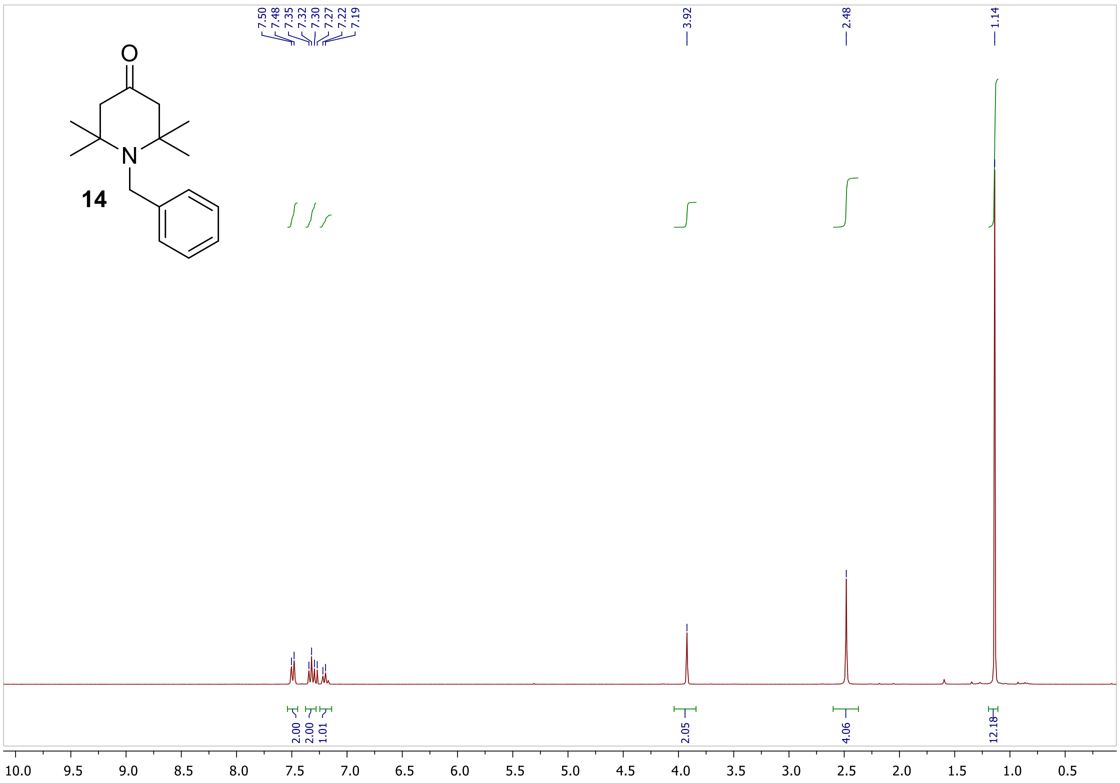

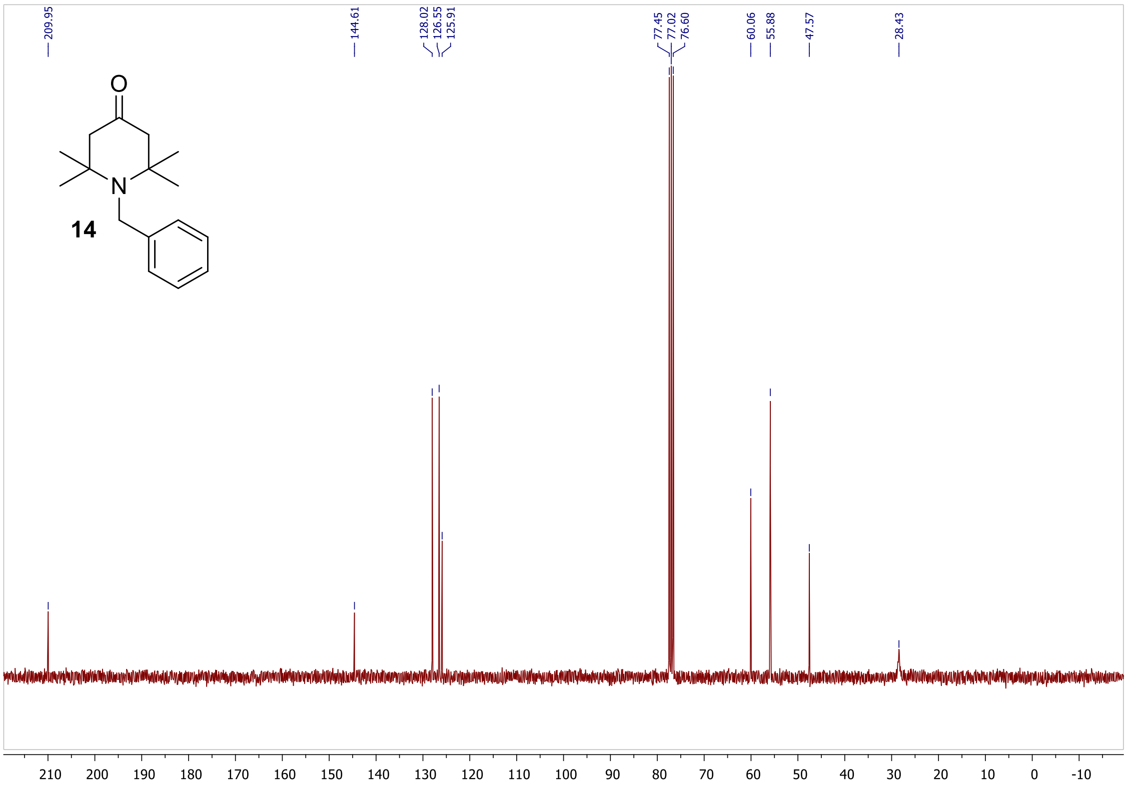

3. Computational work

Unless specified, all calculations were done with Gaussian 09 and the standard defaults are used.[8]
The calculation level is : B3LYP Def2TZVP EmpiricalDispersion=GD3

format : filename E | zpc

NImag is the number of imaginary frequencies
PG is the Point Group
XYZ coordinates are given in standard format

Full references for the Gaussian program.[8]

3.1 Main Structures

3.1.1 TS1_min1 ; label = (A-B)

a/ Pd case

TS1_min1.log -1392.080564 | 0.294063

NImag 0
PG C01 [X(C7H22O4P2Pd1)]

Pd	-0.040693	-0.347328	-0.182744
P	-0.239312	1.892287	-0.013939
P	-2.233615	-0.702127	0.130974
C	-2.424564	-1.855693	1.530344
C	0.415707	2.718410	-1.498099
C	-2.783617	-1.664580	-1.310847
C	0.793571	2.578359	1.322571
O	-3.131329	0.507148	0.348708
O	-1.672423	2.515842	0.224330
H	0.704115	3.666109	1.336157
H	1.836342	2.298924	1.170198
H	0.454995	2.178980	2.277809
H	0.402821	3.801524	-1.363257
H	-0.204090	2.454918	-2.354270
H	1.434609	2.376621	-1.684559
H	-3.486863	-2.075459	1.656181
H	-2.048119	-1.389881	2.439894
H	-1.877946	-2.778094	1.338046
H	-2.386554	1.756557	0.294758
H	-2.762494	-1.024292	-2.191933
H	-3.806211	-2.010420	-1.144533
H	-2.106767	-2.505755	-1.459980
O	0.412417	-2.338352	-0.473043
H	0.309517	-2.881168	0.314565
C	3.315568	-0.263544	0.133515
O	2.180739	-0.494230	-0.719099
C	4.541699	-0.937694	-0.465361
C	3.037120	-0.711482	1.562430
H	3.459773	0.820505	0.115274
H	2.859995	-1.788729	1.594126
H	3.886029	-0.482896	2.209775
H	2.152203	-0.211328	1.958456
H	4.410832	-2.022598	-0.474432
H	5.434762	-0.706644	0.118816
H	1.913621	-1.455833	-0.704042
H	4.697059	-0.602245	-1.490845

b/ Pt case

TS1_min1.log -1383.5502514| 0.294978

NImag 0
PG C01 [X(C7H22O4P2Pt1)]

Pt	-0.048233	-0.303331	-0.164514
P	-0.157859	1.928404	-0.005179
P	-2.246662	-0.580657	0.171137
C	-2.466030	-1.687643	1.603180
C	0.594024	2.729428	-1.454547
C	-2.850417	-1.561653	-1.235948
C	0.826962	2.576903	1.383868
O	-3.102314	0.661621	0.363873
O	-1.573977	2.609141	0.158176
H	0.761604	3.665944	1.405874
H	1.869511	2.275127	1.282046
H	0.431249	2.174527	2.315628
H	0.603137	3.812885	-1.324429
H	0.010312	2.476911	-2.338843
H	1.611440	2.360893	-1.590018
H	-3.533151	-1.870057	1.744062
H	-2.065894	-1.206922	2.494700
H	-1.950951	-2.633853	1.437870
H	-2.315962	1.879682	0.256295
H	-2.809566	-0.946715	-2.134183
H	-3.884290	-1.860754	-1.052128
H	-2.211282	-2.434209	-1.370532
O	0.373567	-2.310194	-0.452816
H	0.232509	-2.844365	0.335083
C	3.285820	-0.249102	0.186527
O	2.174357	-0.506710	-0.696820
C	4.539103	-0.886450	-0.393119
C	2.984613	-0.713126	1.604039
H	3.396524	0.838101	0.173029
H	2.837759	-1.795168	1.625847
H	3.812032	-0.464408	2.271376
H	2.075821	-0.241807	1.979166
H	4.439794	-1.974654	-0.408256
H	5.413335	-0.633384	0.209736
H	1.943094	-1.473515	-0.699208
H	4.705425	-0.541628	-1.413702

3.1.2 TS1 ; label = TS1

a/ Pd case

TS1.log -1392.0744622 | 0.289582

NImag 1
PG C01 [X(C7H22O4P2Pd1)]
36
TS1.log

Pd	-0.027037	-0.342710	-0.171320
P	-0.236002	1.874691	-0.020241
P	-2.237972	-0.675539	0.116404
C	-2.451260	-1.810409	1.529316
C	0.523329	2.661464	-1.470453
C	-2.778650	-1.668309	-1.312620
C	0.724826	2.525970	1.380946
O	-3.150678	0.534761	0.294269
O	-1.673435	2.496691	0.122765
H	0.658481	3.615445	1.392646

H	1.767306	2.220489	1.293871
H	0.312975	2.131420	2.308780
H	0.525259	3.746186	-1.348693
H	-0.048584	2.398618	-2.359279
H	1.542549	2.291758	-1.585524
H	-3.507996	-2.070575	1.619460
H	-2.127632	-1.315605	2.444115
H	-1.861885	-2.714866	1.376897
H	-2.407482	1.733360	0.208504
H	-2.731354	-1.052443	-2.209941
H	-3.809713	-1.992723	-1.156234
H	-2.123910	-2.531264	-1.435788
O	0.613627	-2.395349	-0.428461
H	0.669297	-2.894375	0.393512
C	3.202564	-0.230791	0.118550
O	2.072023	-0.565938	-0.662165
C	4.450280	-0.812082	-0.539679
C	3.059286	-0.693990	1.568265
H	3.295057	0.865300	0.115922
H	2.979861	-1.783705	1.611206
H	3.923055	-0.394659	2.166134
H	2.161876	-0.269706	2.023296
H	4.394109	-1.903419	-0.548766
H	5.355524	-0.516975	-0.003580
H	1.587471	-1.709668	-0.554091
H	4.524510	-0.467644	-1.571408

NORMALMODES:

Pd	0.00	-0.01	0.00
P	0.00	0.00	0.00
P	0.00	0.00	0.00
C	0.00	0.00	0.00
C	0.00	0.00	0.00
C	0.00	0.00	0.00
C	0.00	0.00	0.00
O	0.00	0.00	0.00
O	0.00	0.01	0.00
H	0.00	0.00	0.01
H	0.00	0.01	0.01
H	0.00	0.00	0.00
H	0.01	0.01	-0.01
H	0.00	0.00	0.00
H	0.00	0.01	-0.01
H	0.00	0.00	0.00
H	0.00	0.00	0.00
H	0.01	0.01	-0.01
H	0.00	0.00	0.00
H	0.00	0.00	0.00
H	0.00	0.01	0.01
H	0.01	0.01	0.01
O	-0.02	0.02	0.01
H	-0.11	0.01	0.00
C	0.02	-0.02	0.01
O	0.00	-0.03	-0.01
C	0.01	0.00	0.00
C	0.00	0.00	0.00
H	-0.07	-0.03	-0.05
H	0.00	0.00	0.01
H	0.00	0.01	-0.01
H	0.00	0.00	0.00
H	0.01	0.00	0.00
H	0.00	0.00	0.00
H	0.60	0.78	-0.06
H	0.00	0.00	0.00

-897.8434

b/ Pt case

TS1.log -1383.5428964| 0.290094

NImag 1
PG C01 [X(C7H22O4P2Pt1)]
36
TS1_x_GB.log

Pt	-0.026711	-0.290461	-0.153023
P	-0.193799	1.920230	-0.009909
P	-2.239760	-0.592934	0.151288
C	-2.479585	-1.691060	1.587971
C	0.615243	2.700408	-1.434576
C	-2.826109	-1.597995	-1.250338
C	0.729495	2.550674	1.424115
O	-3.128717	0.638958	0.313290
O	-1.623090	2.559185	0.088142
H	0.672586	3.640287	1.443775
H	1.771383	2.235847	1.371558
H	0.281465	2.150860	2.332756
H	0.620088	3.784772	-1.312203
H	0.066309	2.440501	-2.338548
H	1.635258	2.325972	-1.522231
H	-3.542857	-1.914684	1.693875
H	-2.129801	-1.186702	2.487862
H	-1.922310	-2.618830	1.453552
H	-2.373939	1.801628	0.195158
H	-2.764671	-1.001389	-2.159747
H	-3.865418	-1.886012	-1.080397
H	-2.201592	-2.484477	-1.363774
O	0.596167	-2.362207	-0.399978
H	0.621460	-2.846705	0.432904
C	3.189148	-0.200931	0.160017
O	2.071992	-0.564427	-0.635793
C	4.450045	-0.764161	-0.484925
C	3.033709	-0.658959	1.608399
H	3.256573	0.895827	0.148146
H	2.970662	-1.749608	1.655261
H	3.886647	-0.344356	2.213774
H	2.124737	-0.246686	2.049827
H	4.414155	-1.856343	-0.486976
H	5.344853	-0.449450	0.057035
H	1.591595	-1.719181	-0.527744
H	4.527170	-0.424653	-1.518016

NORMALMODES:

Pt	0.00	0.00	0.00
P	0.00	0.00	0.00
P	0.00	0.00	0.00
C	0.00	0.00	0.00
C	0.00	0.01	0.00
C	0.00	0.00	0.00
C	0.00	0.00	0.00
O	0.00	0.00	0.00
O	0.00	0.01	0.00
H	0.01	0.00	0.01
H	0.00	0.01	0.01
H	0.00	0.00	0.00
H	0.01	0.01	-0.01
H	0.00	0.00	0.00
H	0.00	0.01	-0.01
H	0.00	0.01	-0.01
H	0.00	0.00	0.00
H	0.01	0.01	-0.01

H 0.01 0.01 0.00
H 0.00 0.00 0.00
H 0.00 0.01 0.01
H 0.01 0.01 0.01
O -0.02 0.03 0.01
H -0.10 0.02 0.00
C 0.02 -0.02 0.01
O 0.00 -0.03 -0.01
C 0.01 0.00 0.00
C 0.00 0.00 0.00
H -0.07 -0.03 -0.05
H 0.00 0.00 0.01
H 0.00 0.01 -0.01
H 0.00 0.00 0.00
H 0.01 0.00 0.00
H 0.00 0.00 0.00
H 0.60 0.78 -0.05
H 0.00 0.00 0.00
-909.7299

3.1.3 TS1_TP_min1 ; label = I

a/ Pd case

TS1_TP_min1.log -1392.0773776 | 0.292649

NImag 0
PG C01 [X(C7H22O4P2Pd1)]
Pd -0.049251 -0.391875 -0.147628
P -0.034544 1.830361 -0.035299
P -2.303083 -0.477504 0.096316
C -2.711884 -1.501270 1.552608
C 0.927617 2.479526 -1.428843
C -2.939885 -1.467467 -1.297748
C 0.851126 2.403853 1.445810
O -3.086941 0.837212 0.188086
O -1.407105 2.581123 -0.043434
H 0.899919 3.494335 1.433507
H 1.857428 1.988588 1.472628
H 0.305291 2.079881 2.330839
H 1.068244 3.555859 -1.314410
H 0.384407 2.281638 -2.351798
H 1.888871 1.968347 -1.469538
H -3.796598 -1.607215 1.620571
H -2.345458 -1.011271 2.453862
H -2.254915 -2.487831 1.466260
H -2.242293 1.885352 0.058410
H -2.801170 -0.905176 -2.220573
H -4.005476 -1.657200 -1.152416
H -2.396401 -2.410018 -1.370285
O 0.359676 -2.604324 -0.368107
H 0.395118 -3.078368 0.470747
C 3.110231 -0.313388 0.116609
O 1.970559 -0.710837 -0.592182
C 4.350164 -0.814626 -0.622661
C 3.087075 -0.808075 1.565509
H 3.168852 0.789697 0.141539
H 3.069475 -1.901894 1.583845
H 3.965428 -0.473502 2.123609
H 2.193816 -0.446140 2.079686
H 4.344127 -1.906987 -0.659655
H 5.269877 -0.488720 -0.130114
H 1.276624 -2.190376 -0.506029
H 4.348601 -0.442283 -1.647662

b/ Pt case

not found

3.1.4 TS1_TP ; label = TS_HT

TS1_TP.log -1392.0772542 | 0.290554

NImag 0
PG C01 [X(C7H22O4P2Pd1)]
36
TS1_TP_bis.log

Pd	-0.044703	-0.393790	-0.145047
P	-0.041091	1.833760	-0.036256
P	-2.295606	-0.479819	0.093959
C	-2.737257	-1.479757	1.555122
C	0.926638	2.463869	-1.436328
C	-2.955861	-1.459684	-1.294391
C	0.859605	2.396314	1.441245
O	-3.079674	0.848313	0.176892
O	-1.411245	2.562787	-0.037967
H	0.911863	3.486761	1.429904
H	1.864553	1.978119	1.462999
H	0.316393	2.074342	2.328615
H	1.073969	3.540072	-1.328209
H	0.379455	2.265352	-2.356843
H	1.884350	1.946388	-1.476654
H	-3.823522	-1.574168	1.612455
H	-2.374402	-0.984914	2.455130
H	-2.289001	-2.471523	1.483456
H	-2.293630	1.813797	0.064437
H	-2.806403	-0.904336	-2.219690
H	-4.024402	-1.630319	-1.148060
H	-2.429031	-2.411999	-1.363176
O	0.372121	-2.617788	-0.367305
H	0.415905	-3.090375	0.471989
C	3.109912	-0.304874	0.118444
O	1.969198	-0.702358	-0.588994
C	4.348000	-0.804433	-0.625053
C	3.090466	-0.802568	1.566245
H	3.168057	0.797902	0.144828
H	3.074603	-1.896453	1.582372
H	3.969561	-0.467727	2.122995
H	2.197885	-0.442909	2.083060
H	4.342387	-1.896728	-0.664114
H	5.268725	-0.479043	-0.134121
H	1.283431	-2.197802	-0.508178
H	4.343815	-0.430038	-1.649290

NORMALMODES:

Pd	0.00	0.00	0.00
P	0.04	-0.04	0.00
P	-0.04	0.03	0.00
C	0.03	-0.02	0.00
C	-0.02	0.02	0.01
C	0.03	-0.02	-0.01
C	-0.02	0.02	0.00
O	-0.02	-0.08	0.00
O	-0.02	0.06	0.00

H 0.01 0.02 0.02
H -0.02 0.00 -0.03
H 0.00 0.00 0.00
H 0.01 0.02 -0.01
H 0.00 0.00 0.00
H -0.03 0.00 0.03
H 0.02 0.01 -0.02
H 0.01 -0.01 0.00
H 0.01 -0.04 0.04
H 0.64 0.74 -0.10
H 0.00 0.00 0.00
H 0.02 0.01 0.02
H 0.00 -0.03 -0.04
O 0.00 0.02 0.00
H -0.03 0.01 0.00
C 0.00 0.00 0.00
O 0.01 0.00 0.00
C 0.00 0.00 0.00
C 0.00 0.00 0.00
H 0.01 0.00 0.00
H 0.00 0.00 0.00
H 0.00 0.00 0.00
H 0.00 0.00 0.00
H 0.00 0.00 0.00
H 0.01 0.00 0.00
H 0.01 0.00 0.01
H 0.00 0.00 0.00
-624.6202

b/ Pt case

not found

3.1.5 TS1_min2 ; label = C

a/ Pd case

TS1_min2.log -1392.0792203 | 0.293850

NImag 0
PG C01 [X(C7H22O4P2Pd1)]

Pd	-0.042246	-0.376891	-0.146408
P	-0.029769	1.867034	-0.036436
P	-2.282352	-0.490688	0.091135
C	-2.779764	-1.464583	1.547195
C	1.003130	2.427705	-1.424044
C	-2.995608	-1.425534	-1.297743
C	0.886732	2.361171	1.459971
O	-3.118656	0.845498	0.190755
O	-1.369658	2.587518	-0.056902
H	0.988462	3.448504	1.458542
H	1.870883	1.898237	1.494103
H	0.314010	2.060539	2.336257
H	1.186978	3.499343	-1.323595
H	0.465622	2.242293	-2.353051
H	1.940228	1.873794	-1.440574
H	-3.868105	-1.538418	1.586912
H	-2.419762	-0.974168	2.450660
H	-2.348261	-2.464346	1.485723
H	-2.486888	1.671627	0.087889
H	-2.839783	-0.863634	-2.217949
H	-4.066056	-1.571495	-1.141193
H	-2.493217	-2.389565	-1.383858
O	0.380087	-2.644477	-0.396477

H	0.450919	-3.119132	0.439847
C	3.098699	-0.321839	0.138688
O	1.956299	-0.676374	-0.589824
C	4.331159	-0.817301	-0.616829
C	3.063186	-0.870369	1.567640
H	3.175708	0.777387	0.203646
H	3.030210	-1.963822	1.544898
H	3.944728	-0.569695	2.139711
H	2.174046	-0.514402	2.092797
H	4.307248	-1.907353	-0.693744
H	5.254695	-0.524886	-0.110809
H	1.275036	-2.204253	-0.544195
H	4.338640	-0.407572	-1.627395

b/ Pt case

TS1_min2.log -1383.548252| 0.294584

NImag 0
PG C01 [X(C7H22O4P2Pt1)]

Pt	-0.032320	-0.310652	-0.145897
P	-0.027395	1.927696	-0.033679
P	-2.254659	-0.439814	0.119341
C	-2.728562	-1.198326	1.705011
C	1.068485	2.509675	-1.362504
C	-2.964126	-1.565583	-1.118400
C	0.805152	2.416931	1.511933
O	-3.115382	0.880983	0.025149
O	-1.366490	2.640533	-0.120252
H	0.896748	3.504640	1.530360
H	1.790245	1.961063	1.594165
H	0.189448	2.098186	2.352153
H	1.234930	3.582046	-1.244713
H	0.576475	2.325966	-2.316644
H	2.012859	1.968644	-1.342232
H	-3.815385	-1.273608	1.769552
H	-2.361909	-0.576181	2.520697
H	-2.287908	-2.192436	1.790370
H	-2.489416	1.710928	-0.052787
H	-2.824836	-1.130705	-2.107353
H	-4.030164	-1.705646	-0.931856
H	-2.445414	-2.523787	-1.080339
O	0.380318	-2.599858	-0.392812
H	0.432702	-3.047775	0.460469
C	3.079972	-0.271845	0.202961
O	1.967761	-0.635132	-0.577840
C	4.342307	-0.724968	-0.524637
C	3.002030	-0.855244	1.614573
H	3.125002	0.825595	0.293968
H	2.987994	-1.948253	1.564524
H	3.859118	-0.555616	2.222909
H	2.089642	-0.524930	2.115193
H	4.344353	-1.813234	-0.626077
H	5.243271	-0.425307	0.016100
H	1.290639	-2.210860	-0.555970
H	4.372196	-0.292160	-1.525064

3.1.6 TS2_min1 ; label = Cβ

a/ Pd case

TS2_H2O_min1.log - -1392.06785909| 0.292726
NImag 0

PG C01 [X(C7H22O3P2Pd1H2O)]

Pd	0.068931	-0.331889	-0.083984
P	-2.103228	-0.919901	0.169950
P	-0.463622	1.857743	-0.322748
O	-3.186796	0.022722	-0.501425
C	-2.627780	-1.108493	1.904780
C	-2.446956	-2.557248	-0.548152
O	-1.866382	2.130559	-0.831270
C	0.787540	2.554392	-1.434008
C	-0.172667	2.656589	1.280741
H	-2.523766	-0.149668	2.411924
H	-1.996346	-1.841410	2.409233
H	-3.671088	-1.426281	1.949457
H	-2.209467	-2.539034	-1.611061
H	-1.834330	-3.315524	-0.058586
H	-3.502847	-2.802163	-0.420223
H	0.715953	2.075763	-2.408799
H	1.776978	2.388554	-1.011695
H	0.590834	3.623933	-1.536170
H	-0.377416	3.724177	1.167476
H	-0.855828	2.242693	2.021403
H	0.861984	2.504515	1.590020
H	-2.768904	0.944291	-0.705623
C	2.318595	-1.500551	-0.145074
O	2.094451	-0.142628	-0.342561
C	3.153500	-1.769012	1.106222
C	2.892200	-2.157575	-1.399078
H	1.312545	-2.029369	0.040353
H	3.002037	-3.237520	-1.276559
H	2.245647	-1.958078	-2.253781
H	3.874184	-1.726957	-1.606072
H	2.698730	-1.290593	1.973584
H	4.148928	-1.340918	0.968716
H	3.258899	-2.838895	1.298527
O	3.016048	1.792956	1.282758
H	3.923353	2.020577	1.062234
H	2.776674	1.033664	0.698264

b/ Pt case

TS2_H2O_min1_x_GB E=-1383.53409810 ZPC=0.293468

NImag 0

PG C01 [X(C7H20O3P2Pt1)]

Pt	0.046156	-0.273857	-0.067985
P	-2.091047	-0.905750	0.174364
P	-0.525610	1.894577	-0.305030
O	-3.193504	-0.036626	-0.566877
C	-2.644890	-1.017514	1.904277
C	-2.369935	-2.582862	-0.469784
O	-1.933350	2.143340	-0.797562
C	0.707045	2.599294	-1.430910
C	-0.229105	2.691906	1.295699
H	-2.583002	-0.028310	2.356911
H	-1.997517	-1.696970	2.460290
H	-3.677393	-1.368068	1.948988
H	-2.105898	-2.606371	-1.526192
H	-1.749156	-3.298913	0.070353
H	-3.420924	-2.852650	-0.355000
H	0.626568	2.116791	-2.403062
H	1.703632	2.447072	-1.020236
H	0.497296	3.666057	-1.533382
H	-0.461621	3.754278	1.189781
H	-0.890102	2.257321	2.044190
H	0.813176	2.563231	1.588576
H	-2.821229	0.900408	-0.744162

C 2.377054 -1.439554 -0.111570
O 2.076623 -0.084914 -0.313607
C 3.220924 -1.642732 1.141847
C 2.992960 -2.048635 -1.365398
H 1.406340 -2.015856 0.069551
H 3.180749 -3.117979 -1.245471
H 2.331752 -1.894337 -2.218099
H 3.940983 -1.547892 -1.573601
H 2.726548 -1.198386 2.005550
H 4.183045 -1.142547 1.010278
H 3.403528 -2.701673 1.336593
O 2.996350 1.929056 1.225393
H 3.884809 2.183515 0.961532
H 2.764868 1.143481 0.675724

3.1.7 TS2_H2O ; label = TSβ

a/ Pd case

TS2_H2O -1392.05322526 | 0.287894
NImag 1
PG=C01 [X(C7H22O4P2Pt1)]
36
TS2_H2O.log
Pd 0.181252 -0.308394 -0.117305
P -1.888876 -1.167093 0.184008
P -0.650796 1.858463 -0.292546
O -3.050106 -0.267523 -0.210114
C -2.067897 -1.671609 1.929719
C -2.032343 -2.746928 -0.720210
O -2.108876 1.973016 -0.921165
C 0.369401 2.935759 -1.334598
C -0.703949 2.730810 1.301695
H -1.983116 -0.793623 2.568963
H -1.289708 -2.385683 2.200433
H -3.053635 -2.120271 2.070880
H -1.875524 -2.566791 -1.783072
H -1.287527 -3.458408 -0.363878
H -3.038538 -3.144851 -0.572319
H 0.488134 2.488474 -2.320231
H 1.345706 3.042478 -0.861152
H -0.107926 3.912625 -1.430913
H -1.082315 3.744618 1.155117
H -1.372627 2.195178 1.975575
H 0.301096 2.759418 1.723836
H -2.626828 1.113363 -0.708641
C 2.447489 -1.205783 -0.217775
O 2.333537 0.044151 -0.459029
C 2.991582 -1.628128 1.130561
C 2.727323 -2.126991 -1.385341
H 0.801983 -1.837147 0.053728
H 2.510573 -3.166972 -1.148070
H 2.155810 -1.816414 -2.256711
H 3.793663 -2.037755 -1.619684
H 2.619387 -0.970464 1.913179
H 4.081855 -1.530267 1.085481
H 2.748619 -2.662922 1.364446
O 2.593873 2.172204 1.310224
H 3.476922 2.551613 1.283745
H 2.614439 1.401813 0.709713

NORMALMODES:
Pd 0.01 0.02 -0.01

P 0.00 0.01 0.00
P -0.01 -0.02 0.00
O 0.00 0.00 0.00
C 0.00 0.00 0.00
C 0.02 0.00 0.00
O 0.00 -0.02 0.00
C 0.01 -0.03 0.00
C 0.01 -0.02 0.00
H -0.01 0.00 0.00
H 0.00 0.00 0.00
H 0.00 -0.01 0.00
H -0.01 0.00 0.00
H 0.03 0.00 -0.01
H 0.03 -0.01 0.02
H 0.01 -0.02 0.00
H 0.00 -0.03 0.01
H -0.01 -0.04 0.02
H 0.01 -0.02 -0.02
H 0.00 0.00 0.01
H 0.01 -0.02 -0.01
H -0.01 -0.02 0.01
C -0.19 -0.09 0.04
O -0.06 0.00 0.01
C 0.02 -0.01 0.00
C 0.03 -0.01 0.00
H 0.90 0.21 -0.15
H 0.10 -0.04 -0.07
H 0.04 -0.03 -0.02
H 0.03 0.09 0.04
H 0.02 -0.03 0.02
H 0.01 0.07 -0.08
H 0.11 -0.03 0.06
O 0.00 -0.01 -0.01
H 0.00 -0.02 0.00
H 0.00 -0.03 0.01
-439.0664

b/ Pt case

TS2_H2O_x_GB E=-1383.52284083 ZPC=0.288861

NImag 1
PG C01 [X(C7H20O3P2Pt1)]
36
TS2_H2O_x_GB.log
Pt 0.145377 -0.254518 -0.097658
P -1.915668 -1.117361 0.207116
P -0.697219 1.873905 -0.287706
O -3.090367 -0.228178 -0.170113
C -2.083555 -1.639048 1.947162
C -2.039238 -2.687943 -0.713598
O -2.144890 1.982566 -0.927787
C 0.340376 2.933868 -1.323707
C -0.758414 2.728747 1.310806
H -2.005317 -0.764328 2.591643
H -1.297137 -2.346386 2.211656
H -3.063450 -2.099464 2.088680
H -1.901756 -2.488608 -1.775665
H -1.275944 -3.390519 -0.379117
H -3.033848 -3.109926 -0.556779
H 0.464888 2.476311 -2.303711
H 1.312011 3.038795 -0.840715
H -0.131147 3.912012 -1.432087
H -1.144154 3.740611 1.171544
H -1.424679 2.181081 1.977009

H 0.245522 2.761110 1.735073
H -2.671061 1.127181 -0.695046
C 2.427640 -1.139991 -0.187004
O 2.310987 0.125666 -0.421792
C 2.994022 -1.552550 1.154805
C 2.751651 -2.030000 -1.366921
H 0.893806 -1.774365 0.059050
H 2.573267 -3.081634 -1.147797
H 2.171635 -1.726504 -2.235134
H 3.814330 -1.895062 -1.593425
H 2.594237 -0.919083 1.943612
H 4.078462 -1.407491 1.108493
H 2.794829 -2.599218 1.379050
O 2.555376 2.254404 1.337328
H 3.434605 2.641645 1.300007
H 2.581040 1.473939 0.749496

NORMALMODES:
Pt 0.01 0.02 0.00
P 0.00 0.00 0.00
P -0.01 -0.02 0.00
O 0.00 0.00 0.00
C 0.00 0.00 0.00
C 0.01 0.00 0.00
O 0.00 -0.02 0.01
C 0.01 -0.03 0.01
C 0.01 -0.02 0.00
H 0.00 0.00 0.00
H 0.00 0.00 0.00
H 0.00 -0.01 0.01
H -0.01 0.00 0.00
H 0.01 -0.01 -0.01
H 0.01 -0.02 0.01
H 0.01 -0.02 0.00
H 0.00 -0.03 0.01
H -0.02 -0.04 0.03
H 0.01 -0.02 -0.03
H 0.00 0.00 0.01
H 0.01 -0.02 0.00
H -0.01 -0.03 0.02
C -0.18 -0.11 0.04
O -0.06 0.00 0.01
C 0.03 -0.01 0.00
C 0.04 -0.01 -0.01
H 0.91 0.23 -0.11
H 0.10 -0.04 -0.07
H 0.04 -0.02 -0.01
H 0.03 0.08 0.04
H 0.03 -0.03 0.01
H 0.02 0.06 -0.06
H 0.11 -0.02 0.06
O 0.00 -0.01 -0.01
H 0.00 -0.02 0.00
H 0.00 -0.03 0.00

3.1.1 -476.0256TS2_min2 ; label = Dβ

a/ Pd case

TS2_H2O_min2 -1392.05752780 | 0.288142
NImag 0
PG C01 [X(C7H22O3P2Pd1H2O)]
Pd 0.003738 -0.427935 -0.077963
P -2.183494 -0.832665 0.193327

P	-0.266262	1.924310	-0.300132
O	-3.084021	0.340615	-0.148794
C	-2.482602	-1.354716	1.915374
C	-2.702170	-2.277047	-0.792349
O	-1.696710	2.362323	-0.868045
C	0.895912	2.742682	-1.435489
C	-0.041217	2.865364	1.243663
H	-2.184581	-0.552599	2.589352
H	-1.903760	-2.248937	2.144593
H	-3.549705	-1.549300	2.043781
H	-2.474948	-2.093084	-1.841612
H	-2.173280	-3.170579	-0.463077
H	-3.780896	-2.404344	-0.678663
H	0.845314	2.269864	-2.415324
H	1.905820	2.638663	-1.038880
H	0.644423	3.800751	-1.528029
H	-0.196359	3.930544	1.059128
H	-0.772691	2.520950	1.975045
H	0.965521	2.691838	1.626823
H	-2.368586	1.630496	-0.652888
C	2.541565	-1.472245	-0.274915
O	2.350142	-0.276948	-0.528401
C	2.970179	-1.917532	1.095141
C	2.539705	-2.491705	-1.378907
H	0.045253	-2.030301	0.129540
H	2.119114	-3.438895	-1.046955
H	1.992911	-2.114442	-2.239082
H	3.582096	-2.668403	-1.667448
H	2.865865	-1.107995	1.813721
H	4.020996	-2.222591	1.043744
H	2.392329	-2.784474	1.412158
O	3.110910	1.647096	1.401795
H	3.946009	2.044499	1.137428
H	2.882739	1.015168	0.697469

b/ Pt case

TS2_H2O_min2_x_GB E=-1383.53571158 ZPC=0.288749

NImag 0
PG C01 [X(C7H20O3P2Pt1)]

Pt	-0.056960	-0.418654	0.131347
P	-2.268279	-0.681333	-0.026355
P	-0.120095	1.842396	-0.513557
O	-3.016131	0.454018	-0.707878
C	-2.965386	-0.953883	1.635927
C	-2.638173	-2.236463	-0.904140
O	-1.381011	2.257302	-1.394885
C	1.295705	2.356318	-1.538504
C	-0.069426	3.028976	0.865289
H	-2.806158	-0.060580	2.238862
H	-2.480974	-1.803198	2.116728
H	-4.038850	-1.132526	1.545249
H	-3.721929	-2.339376	-0.988399
H	-2.207382	-2.188766	-1.903551
H	-2.214740	-3.086926	-0.371781
H	1.338730	1.733136	-2.431570
H	2.217192	2.232003	-0.968036
H	1.189958	3.400649	-1.836647

H	-0.976181	2.899956	1.457126
H	0.790265	2.823722	1.504990
H	-0.033827	4.052866	0.488291
H	-2.138770	1.586839	-1.213050
C	3.035181	-0.984456	-0.365625
O	2.232682	-0.336343	0.298563
C	4.495384	-0.922543	-0.044562
C	2.569458	-1.849995	-1.493396
H	-0.026987	-1.989316	0.580586
H	1.845989	-2.565358	-1.092100
H	2.018661	-1.232122	-2.205761
H	3.384832	-2.367120	-1.995039
H	5.049707	-0.584197	-0.924294
H	4.857369	-1.928962	0.184014
H	4.683781	-0.259242	0.796129
O	1.699708	0.916720	2.918079
H	2.118930	0.518282	2.144215
H	0.800458	0.567994	2.886638

3.1.2 TS1-2_min1 ; label = (A-B)'

a/ Pd case

TS1-2_min1.log -1392.0708194 | 0.293755

NImag 0

PG C01 [X(C7H22O4P2Pd1)]

Pd	0.046140	-0.327809	-0.191621
P	2.252488	-0.646679	0.128890
P	0.176327	1.929759	-0.022236
C	-0.863096	2.603154	1.313074
C	2.873676	-1.643138	-1.260597
C	-0.487609	2.754343	-1.504467
C	2.348596	-1.746520	1.570738
O	1.600180	2.588960	0.204831
O	3.111149	0.587371	0.310034
H	1.692809	-2.601651	1.408769
H	2.028950	-1.201652	2.457962
H	3.384866	-2.066239	1.702163
H	3.938814	-1.823202	-1.099698
H	2.742251	-1.095230	-2.192226
H	2.339387	-2.590643	-1.308851
H	-0.769535	3.690297	1.337281
H	-0.537778	2.192021	2.267801
H	-1.905032	2.328963	1.146404
H	2.326147	1.863172	0.269550
H	0.110789	2.474112	-2.370468
H	-0.448015	3.837418	-1.374730
H	-1.519569	2.443037	-1.671957
C	-2.967008	-0.685981	0.054898
O	-2.960455	-1.948096	-0.539514
C	-2.978636	-0.770222	1.579353
C	-4.128519	0.117773	-0.506386
H	-2.031789	-0.119151	-0.246954
H	-5.066940	-0.380436	-0.254329
H	-4.150318	1.127439	-0.090698
H	-4.056435	0.178495	-1.592425
H	-3.893772	-1.269176	1.904022
H	-2.936502	0.219762	2.039501
H	-2.127460	-1.354645	1.930979
O	-0.301021	-2.327788	-0.338607
H	-2.046139	-2.315936	-0.435262
H	-0.012616	-2.683734	-1.185691

b/ Pt case

TS1-2_min1.log -1383.5400406| 0.294278

NImag 0
PG C01 [X(C7H22O4P2Pt1)]

Pt	0.054736	-0.288452	-0.130639
P	2.274970	-0.536975	0.126009
P	0.117973	1.953838	-0.014925
C	-0.810997	2.612833	1.403666
C	2.856291	-1.605725	-1.225874
C	-0.711244	2.719026	-1.441697
C	2.463962	-1.546346	1.624134
O	1.529133	2.672370	0.056853
O	3.111223	0.720677	0.194283
H	1.835745	-2.433017	1.542063
H	2.157034	-0.960828	2.489508
H	3.514491	-1.824267	1.733143
H	3.930569	-1.757741	-1.103601
H	2.670675	-1.117115	-2.180939
H	2.340052	-2.564387	-1.193910
H	-0.745851	3.702026	1.411947
H	-0.385684	2.217794	2.325269
H	-1.855857	2.308829	1.337802
H	2.283149	1.979668	0.119378
H	-0.179456	2.444534	-2.351796
H	-0.709251	3.805174	-1.336051
H	-1.738382	2.358288	-1.510743
C	-2.971705	-0.655033	0.073432
O	-2.988394	-1.910818	-0.528354
C	-3.001452	-0.742981	1.595404
C	-4.091308	0.190903	-0.507024
H	-2.008723	-0.112145	-0.214462
H	-5.051609	-0.273425	-0.273166
H	-4.083542	1.199036	-0.087995
H	-3.996588	0.251127	-1.591225
H	-3.933742	-1.217238	1.908370
H	-2.936945	0.244071	2.058119
H	-2.168926	-1.349408	1.952975
O	-0.324978	-2.296731	-0.244256
H	-2.094067	-2.308052	-0.390216
H	-0.023805	-2.666666	-1.081318

3.1.3 TS1-2 ; label = TS'

a/ Pd case

TS1-2.log -1392.052168 | 0.286967

NImag 1
PG C01 [X(C7H22O4P2Pd1)]
39
TS1-2-H2O.log

Pd	-0.073151	-0.093789	-0.225912
P	2.247194	-0.036561	-0.141248
P	-0.235208	2.117636	0.150375
C	-1.039028	2.397527	1.764525
C	3.069828	-1.255623	-1.206763
C	-1.423056	2.815859	-1.046934
C	2.872199	-0.429526	1.519723
O	1.056265	2.918964	0.086799

O	2.907306	1.345171	-0.565079
H	2.530305	-1.425634	1.803040
H	2.488298	0.309630	2.222752
H	3.963575	-0.391494	1.519849
H	4.150109	-1.107813	-1.155376
H	2.743034	-1.126217	-2.238121
H	2.823447	-2.257925	-0.854100
H	-1.129745	3.473457	1.927891
H	-0.423761	1.968960	2.554686
H	-2.024852	1.933628	1.787939
H	2.248331	2.107512	-0.347458
H	-1.040769	2.663707	-2.055571
H	-1.512041	3.888223	-0.859931
H	-2.395911	2.335061	-0.958022
C	-2.943949	-1.122188	-0.032099
O	-2.686863	-2.130061	-0.759398
C	-2.863642	-1.294558	1.478165
C	-4.008237	-0.160933	-0.530392
H	-1.768773	-0.101246	-0.179731
H	-4.977787	-0.656118	-0.413785
H	-4.026829	0.769008	0.037434
H	-3.857773	0.048905	-1.587351
H	-3.748232	-1.859815	1.790156
H	-2.851409	-0.342735	2.007083
H	-1.979729	-1.870544	1.747138
O	-0.231427	-2.273221	-0.745057
H	-1.279252	-2.376755	-0.751561
H	0.073622	-2.451435	-1.642100
O	1.582646	-3.444965	1.028466
H	1.337951	-4.303836	1.384617
H	0.835250	-3.158810	0.472386

NORMALMODES:

Pd	-0.01	-0.02	0.00
P	-0.02	0.02	0.00
P	0.01	0.00	0.00
C	0.00	-0.01	0.00
C	-0.05	0.00	0.00
C	0.00	-0.03	0.00
C	-0.04	0.00	0.00
O	0.01	0.00	0.00
O	-0.03	0.00	0.00
H	-0.03	0.00	-0.01
H	-0.02	0.00	0.01
H	-0.04	0.00	-0.04
H	-0.05	0.04	0.03
H	-0.03	0.00	0.00
H	-0.04	0.01	0.01
H	-0.02	-0.01	-0.01
H	0.00	0.01	0.01
H	0.01	-0.03	0.00
H	-0.03	0.03	0.02
H	0.01	0.00	0.00
H	-0.03	-0.04	0.03
H	0.00	-0.05	-0.01
C	0.29	0.21	-0.02
O	0.08	-0.03	-0.01
C	-0.02	0.00	0.01
C	0.02	-0.01	0.00
H	-0.42	-0.66	0.10
H	0.10	-0.15	0.04
H	-0.09	0.01	-0.02
H	-0.05	0.01	-0.01
H	-0.05	-0.03	-0.15
H	-0.07	-0.04	0.11

H -0.04 -0.01 0.07
O -0.06 0.05 0.02
H -0.30 0.12 0.00
H -0.06 0.00 0.03
O -0.01 0.01 -0.01
H -0.01 0.02 0.00
H -0.02 0.01 -0.02
-417.8582

b/ Pt case

TS1-2.log -1383.5223432| 0.286325

NImag 1
PG C01 [X(C7H22O4P2Pt1)]
36
TS_Di_x_GB.log

Pt	-0.019172	-0.236559	-0.081042
P	2.250814	-0.634131	0.072323
P	0.206183	1.968881	-0.001939
C	-0.701580	2.703870	1.390175
C	2.777271	-1.729424	-1.290562
C	-0.533653	2.759998	-1.461430
C	2.537174	-1.655634	1.554657
O	1.664413	2.544018	0.100070
O	3.160503	0.593518	0.102106
H	1.894447	-2.536325	1.530748
H	2.299684	-1.065366	2.439067
H	3.586578	-1.954856	1.596797
H	3.849520	-1.915529	-1.202546
H	2.578039	-1.242999	-2.244604
H	2.243179	-2.679846	-1.247378
H	-0.548331	3.784324	1.391841
H	-0.327721	2.285530	2.323478
H	-1.764313	2.480361	1.299903
H	2.398015	1.768830	0.107380
H	-0.014376	2.415328	-2.354596
H	-0.430941	3.843603	-1.381447
H	-1.586593	2.488685	-1.536116
C	-3.021978	-0.754633	0.051961
O	-2.888218	-1.950908	-0.385378
C	-3.133827	-0.574143	1.559745
C	-3.871873	0.184277	-0.788644
H	-1.751094	-0.023894	-0.149947
H	-4.916585	-0.123610	-0.680440
H	-3.782548	1.222725	-0.467839
H	-3.596873	0.094513	-1.837866
H	-4.118986	-0.939809	1.865855
H	-3.035034	0.467391	1.864580
H	-2.376119	-1.172332	2.063044
O	-0.498704	-2.347323	-0.187772
H	-1.577369	-2.333804	-0.234022
H	-0.195296	-2.752859	-1.008001

NORMALMODES:
Pt 0.00 -0.02 0.00
P -0.02 0.01 0.00
P 0.00 0.00 0.00
C 0.00 0.00 0.00
C -0.04 0.01 0.00
C 0.00 0.00 0.00
C -0.05 0.01 0.00
O 0.00 0.03 0.00
O -0.02 -0.02 0.00
H -0.04 0.01 -0.01

H -0.02 0.01 0.00
H -0.04 0.03 -0.05
H -0.04 0.04 0.04
H -0.02 0.01 -0.01
H -0.04 0.01 0.00
H -0.02 0.00 -0.01
H 0.00 0.01 0.00
H 0.00 -0.02 0.01
H -0.03 0.10 0.00
H 0.00 0.00 0.00
H -0.01 0.00 0.00
H 0.00 0.00 0.00
C 0.28 0.16 -0.02
O 0.09 -0.05 0.00
C -0.02 0.01 0.00
C 0.00 -0.02 0.01
H -0.49 -0.47 0.11
H 0.05 -0.14 0.08
H -0.10 0.01 -0.03
H -0.05 0.00 -0.01
H -0.03 -0.05 -0.13
H -0.09 -0.01 0.10
H -0.05 0.01 0.05
O -0.06 0.08 0.00
H -0.46 0.21 -0.10
H -0.05 0.06 0.01
-431.3887

3.1.4 TS1-2_min2 ; label = D'

a/ Pd case

TS1-2_min2.log -1392.0712316 | 0.289830

NImag 0
PG C01 [X(C7H22O4P2Pd1)]

Pd	0.514976	0.077412	1.058439
P	-0.566237	1.571641	-0.456072
P	1.984723	-0.565466	-0.494095
C	2.714596	-2.206096	-0.176001
C	0.246594	3.188865	-0.718691
C	3.455150	0.520098	-0.503989
C	-2.271225	2.084756	-0.076787
O	1.348182	-0.600291	-1.884722
O	-0.702096	0.933770	-1.928530
H	-2.264574	2.672009	0.842585
H	-2.901472	1.211638	0.079326
H	-2.670301	2.688622	-0.893861
H	-0.312067	3.788029	-1.440308
H	1.255028	3.025714	-1.097771
H	0.313245	3.723870	0.230325
H	3.361301	-2.473732	-1.014419
H	1.917039	-2.942141	-0.088193
H	3.281421	-2.189388	0.754266
H	0.086315	0.295092	-2.056514
H	3.154709	1.541277	-0.735179
H	4.154139	0.173696	-1.268594
H	3.935980	0.505916	0.474639
C	-2.457858	-1.660734	-0.361310
O	-2.778123	-1.126141	0.688738
C	-1.283115	-2.588713	-0.467532
C	-3.205502	-1.362170	-1.630789
H	1.324363	-0.944078	1.988772
H	-2.609807	-0.627060	-2.182807

H	-3.304956	-2.246734	-2.260907
H	-4.180864	-0.934718	-1.407942
H	-0.578653	-2.193963	-1.205983
H	-1.618553	-3.564825	-0.828598
H	-0.782676	-2.693075	0.491938
O	-1.228147	0.208290	2.551273
H	-1.862111	-0.350301	2.051431
H	-0.974039	-0.300489	3.328617

b/ Pt case

TS1-2_min2.log -1383.5435288| 0.290830

NImag 0

PG C01 [X(C7H22O4P2Pt1)]

Pt	-0.425160	0.031211	-0.853937
P	0.583315	1.571306	0.602183
P	-1.875568	-0.595235	0.712537
C	-2.490271	-2.283114	0.404838
C	-0.298967	3.159791	0.768161
C	-3.400118	0.405554	0.645341
C	2.265094	2.116916	0.184036
O	-1.283207	-0.552132	2.119465
O	0.735830	1.018795	2.102919
H	2.234067	2.646602	-0.768877
H	2.924838	1.258783	0.075244
H	2.646646	2.780959	0.961362
H	0.227705	3.823237	1.456576
H	-1.300489	2.970287	1.152687
H	-0.385140	3.634382	-0.210553
H	-3.146071	-2.575640	1.227189
H	-1.645756	-2.968865	0.357818
H	-3.026470	-2.321205	-0.542506
H	-0.037864	0.366350	2.262764
H	-3.162818	1.445893	0.864075
H	-4.106241	0.038677	1.393220
H	-3.844111	0.345756	-0.348615
C	2.578918	-1.607714	0.439415
O	2.908704	-1.087647	-0.615225
C	1.441560	-2.581534	0.542074
C	3.279919	-1.251578	1.720316
H	-1.146791	-1.025483	-1.863183
H	2.639010	-0.534249	2.245024
H	3.400749	-2.119384	2.369770
H	4.241577	-0.786478	1.514062
H	0.724703	-2.218639	1.284151
H	1.821271	-3.542927	0.900585
H	0.945116	-2.705150	-0.416396
O	1.299774	0.267010	-2.376611
H	1.960169	-0.303487	-1.921913
H	1.036516	-0.203105	-3.175676

3.2 Additionnal Structures

3.2.1 H2O

H2O.log -76.4630039 | 0.021178

NImag 0
PG C02V [C2(O1), SGV(H2)]

O	0.000000	0.000000	0.116855
H	0.000000	0.765002	-0.467419
H	0.000000	-0.765002	-0.467419

3.2.2 cetone

Acetone.log -193.2426035 | 0.083260

NImag 0
PG C01 [X(C3H6O1)]

C	0.000000	0.186361	0.000002
O	0.000030	1.395092	0.000000
C	-1.286395	-0.612205	-0.000674
H	-2.142126	0.058157	-0.036709
H	-1.310449	-1.292525	-0.855935
H	-1.341466	-1.231667	0.898640
C	1.286370	-0.612245	0.000675
H	1.310382	-1.292617	0.855897
H	1.341458	-1.231645	-0.898680
H	2.142112	0.058100	0.036772

3.2.3 propanol

Isopropanol.log -194.4492354 | 0.107717

NImag 0
PG C01 [X(C3H8O1)]

C	-0.001961	0.038460	0.365325
H	0.006248	0.083864	1.464090
C	-1.325938	-0.548734	-0.089865
H	-1.459837	-1.555011	0.311000
H	-2.153771	0.075265	0.248120
H	-1.357565	-0.599586	-1.180255
C	1.193215	-0.783415	-0.103251
H	1.207033	-0.837815	-1.193812
H	2.132686	-0.333911	0.229715
H	1.150226	-1.798883	0.296949
O	0.049344	1.369686	-0.162265
H	0.888335	1.770727	0.089054

3.3 removing water from the ß elimination path

For the β-elimination mechanism it is about +50 kj/mol higher than keeping H2O

3.3.1 TS2_min1 ; label = Cβ

a/ Pd case

TS2_min1.log -1315.5844406 | 0.267710

NImag 0
PG C01 [X(C7H22O3P2Pd1)]

Pd	0.241644	-0.228841	-0.002344
P	-1.848940	-1.117329	-0.004525
P	-0.456522	1.917068	-0.005678
O	-3.069270	-0.103143	-0.076725
C	-2.200411	-2.148126	1.457939
C	-2.148036	-2.272014	-1.383050
O	-1.951494	2.158516	-0.042959
C	0.387360	2.693680	-1.414826
C	0.310972	2.670506	1.459134
H	-2.115579	-1.535616	2.354866
H	-1.480645	-2.965922	1.519856
H	-3.211627	-2.554159	1.393573
H	-2.028867	-1.741064	-2.326750
H	-1.428934	-3.091759	-1.345264
H	-3.161951	-2.671483	-1.320338
H	-0.030867	2.303392	-2.341477
H	1.450438	2.459195	-1.363699
H	0.226219	3.773363	-1.374369
H	0.147874	3.750270	1.431004
H	-0.151870	2.261624	2.356124
H	1.376474	2.441574	1.457815
H	-2.741567	0.870019	-0.069513
C	2.670415	-0.922908	0.003469
O	2.182217	0.370032	0.005486
C	3.455800	-1.238735	1.277567
C	3.458273	-1.233787	-1.270338
H	1.785980	-1.669784	0.001377
H	3.792531	-2.273556	-1.299532
H	2.844396	-1.031263	-2.148250
H	4.334368	-0.582882	-1.309404
H	2.840145	-1.039920	2.155084
H	4.331672	-0.587805	1.320891
H	3.790254	-2.278535	1.303099

b/ Pt case

TS2_min1.log -1307.0502688| 0.268085

NImag 0
PG C01 [X(C7H20O3P2Pt1)]

Pt	0.202430	-0.165929	-0.000423
P	-1.810594	-1.167289	-0.011211
P	-0.614289	1.927899	-0.013180
O	-3.087800	-0.249429	-0.238698
C	-2.169407	-2.084191	1.521021
C	-1.967427	-2.447491	-1.295269
O	-2.114885	2.085341	-0.085545
C	0.219139	2.750150	-1.401192
C	0.072026	2.717701	1.471368
H	-2.174367	-1.385299	2.356628
H	-1.398289	-2.835915	1.696518
H	-3.144975	-2.568349	1.449787
H	-1.822808	-1.989652	-2.272929
H	-1.208954	-3.218097	-1.149331
H	-2.959706	-2.899504	-1.250797
H	-0.155956	2.336184	-2.335851

H	1.292818	2.577669	-1.328761
H	-0.004643	3.818459	-1.364148
H	-0.167544	3.782930	1.447531
H	-0.377063	2.267881	2.355371
H	1.151237	2.567570	1.491962
H	-2.835413	0.739379	-0.199192
C	2.692447	-0.796747	0.009799
O	2.138389	0.478632	0.009514
C	3.487904	-1.061949	1.284847
C	3.487416	-1.063185	-1.265374
H	1.844401	-1.582219	0.010744
H	3.881626	-2.081566	-1.294123
H	2.857015	-0.898665	-2.139219
H	4.323725	-0.362118	-1.310783
H	2.857992	-0.896278	2.158836
H	4.324312	-0.360899	1.328873
H	3.882074	-2.080314	1.314377

3.3.2 TS2 ; label = TSβ

a/ Pd case

TS2.log -1315.5732161 | 0.262888

NImag 10

PG C01 [X(C7H22O3P2Pd1)]

Pd	0.365587	-0.007981	0.000028
P	-0.993234	1.803030	-0.000023
P	-1.383997	-1.513879	-0.000054
O	-2.494617	1.528929	-0.000237
C	-0.587777	2.865589	-1.428680
C	-0.588164	2.865466	1.428831
O	-2.853452	-0.929976	-0.000718
C	-1.297112	-2.643383	1.424301
C	-1.296188	-2.644207	-1.423685
H	-0.773331	2.313673	-2.349477
H	0.460574	3.163086	-1.395347
H	-1.229977	3.748706	-1.408678
H	-0.773980	2.313489	2.349538
H	0.460199	3.162955	1.395812
H	-1.230350	3.748589	1.408696
H	-1.409588	-2.069793	2.343665
H	-0.325242	-3.138529	1.430984
H	-2.094271	-3.386907	1.364296
H	-2.093269	-3.387814	-1.363689
H	-1.408218	-2.071185	-2.343456
H	-0.324234	-3.139195	-1.429503
H	-2.824673	0.118294	-0.000555
C	2.743502	-0.377358	0.000022
O	1.993745	-1.418236	-0.000022
C	3.472728	-0.028796	-1.286283
C	3.472742	-0.028894	1.286348
H	1.737981	0.955517	0.000075
H	3.842502	0.995709	1.285221
H	2.822632	-0.187269	2.143863
H	4.327924	-0.707258	1.369801
H	2.822581	-0.187060	-2.143791
H	4.327878	-0.707189	-1.369832
H	3.842531	0.995792	-1.285065

b/ Pt case

TS2.log -1307.0421539| 0.263178

NImag 1
PG C01 [X(C7H20O3P2Pt1)]

Pt	0.297297	0.009656	0.000015
P	-1.162125	1.732082	-0.000123
P	-1.350116	-1.571281	-0.000008
O	-2.649057	1.382190	-0.000286
C	-0.818425	2.813514	-1.428735
C	-0.818728	2.813491	1.428580
O	-2.844644	-1.076916	-0.000316
C	-1.174059	-2.680418	1.427522
C	-1.173607	-2.680800	-1.427183
H	-0.985345	2.251330	-2.346860
H	0.214933	3.160529	-1.405525
H	-1.500221	3.665866	-1.404310
H	-0.985848	2.251295	2.346661
H	0.214635	3.160502	1.405597
H	-1.500515	3.665847	1.404020
H	-1.326171	-2.108922	2.342231
H	-0.167158	-3.099379	1.432604
H	-1.913677	-3.481326	1.374189
H	-1.913201	-3.481730	-1.373843
H	-1.325482	-2.109559	-2.342091
H	-0.166683	-3.099706	-1.431858
H	-2.881132	-0.015646	-0.000328
C	2.695774	-0.220159	0.000113
O	2.004117	-1.319267	0.000127
C	3.419351	0.139524	-1.285594
C	3.419264	0.139612	1.285844
H	1.697005	1.003573	0.000040
H	3.763358	1.173547	1.288055
H	2.772872	-0.037190	2.142473
H	4.289901	-0.518416	1.365290
H	2.773017	-0.037335	-2.142255
H	4.289993	-0.518511	-1.364937
H	3.763447	1.173458	-1.287851

3.3.3 TS2_min2 ; label = Dβ

a/ Pd case

TS2_min2.log -1315.5862518 | 0.263205

NImag 0
PG C01 [X(C7H22O3P2Pd1)]

Pd	-0.007733	-0.475631	-0.232159
P	2.194254	-0.764475	0.034800
P	0.025342	1.872590	0.127533
O	2.964855	0.497313	0.410805
C	2.927573	-1.469223	-1.480859
C	2.530233	-2.042455	1.294133
O	1.447495	2.494981	0.478480
C	-1.066434	2.456442	1.473471
C	-0.578327	2.859408	-1.285593
H	2.757077	-0.784225	-2.310678
H	2.469268	-2.430247	-1.712051
H	4.003309	-1.584552	-1.330710
H	2.083830	-1.737732	2.240072
H	2.101264	-2.995593	0.986614
H	3.610896	-2.132938	1.423945
H	-0.769282	1.979559	2.407291
H	-2.100265	2.186947	1.249707

H	-0.989081	3.539718	1.585503
H	-0.548533	3.925507	-1.051519
H	0.056105	2.664659	-2.149911
H	-1.599995	2.561348	-1.526929
H	2.159802	1.742214	0.476352
C	-3.141901	-0.994313	-0.032467
O	-2.252924	-0.351195	-0.571329
C	-4.574801	-0.770771	-0.421575
C	-2.842612	-2.008974	1.031763
H	0.079390	-2.067052	-0.461781
H	-3.673157	-2.693003	1.200666
H	-1.931024	-2.547253	0.766005
H	-2.637208	-1.473900	1.963620
H	-4.657877	0.044752	-1.135903
H	-5.175568	-0.560927	0.467283
H	-4.974963	-1.689460	-0.860136

b/ Pt case

TS2_min2.log -1307.0593829| 0.264323

NImag 0
PG C01 [X(C7H20O3P2Pt1)]

Pt	-0.008019	-0.395412	-0.190031
P	2.189594	-0.669465	0.080232
P	0.034782	1.925288	0.147509
O	2.976380	0.582941	0.449456
C	2.907215	-1.391679	-1.432688
C	2.497799	-1.945153	1.346786
O	1.449666	2.562870	0.481017
C	-1.056411	2.501371	1.492543
C	-0.587949	2.870209	-1.281192
H	2.757293	-0.698321	-2.259444
H	2.424621	-2.339204	-1.669619
H	3.978614	-1.537688	-1.280324
H	2.067968	-1.615493	2.291988
H	2.038684	-2.888614	1.053749
H	3.575815	-2.067969	1.469271
H	-0.742044	2.036598	2.426682
H	-2.086675	2.211439	1.279180
H	-0.997064	3.586555	1.594689
H	-0.582706	3.941085	-1.069035
H	0.052929	2.670540	-2.139460
H	-1.601546	2.542447	-1.517425
H	2.171833	1.813281	0.493296
C	-3.127176	-0.885358	0.027156
O	-2.249131	-0.230835	-0.522114
C	-4.559213	-0.691066	-0.371290
C	-2.801920	-1.889422	1.089877
H	-0.033551	-2.011284	-0.439277
H	-3.685748	-2.388111	1.483106
H	-2.103543	-2.616603	0.666767
H	-2.260293	-1.389620	1.895359
H	-4.644498	0.056578	-1.156048
H	-5.145238	-0.390676	0.501824
H	-4.974405	-1.643356	-0.713010

4. References

1. Gatineau, D.; Nguyen, D.H.; Hérault, D.; Vanthuyne, N.; Leclaire, J.; Giordano, L.; Buono, G. H-Adamantylphosphinates as Universal Precursors of P-Stereogenic Compounds. *J. Org. Chem.* **2015**, *80*, 4132–4141, doi:10.1021/acs.joc.5b00548.
2. Membrat, R.; Vasseur, A.; Martinez, A.; Giordano, L.; Nuel, D. Phosphinous Acid Platinum Complex as Robust Catalyst for Oxidation: Comparison with Palladium and Mechanistic Investigations. *Eur. J. Org. Chem.* **2018**, *2018*, 5427–5434, doi:10.1002/ejoc.201801040.
3. Bigeault, J.; Giordano, L.; Buono, G. [2+1] Cycloadditions of Terminal Alkynes to Norbornene Derivatives Catalyzed by Palladium Complexes with Phosphinous Acid Ligands. *Angew. Chem. Int. Ed.* **2005**, *44*, 4753–4757, doi:10.1002/anie.200500879.
4. Chan, E.Y.Y.; Zhang, Q.-F.; Sau, Y.-K.; Lo, S.M.F.; Sung, H.H.Y.; Williams, I.D.; Haynes, R.K.; Leung, W.-H. Chiral Bisphosphinite Metalloligands Derived from a P-Chiral Secondary Phosphine Oxide. *Inorg. Chem.* **2004**, *43*, 4921–4926, doi:10.1021/ic049744u.
5. Membrat, R.; Vasseur, A.; Giordano, L.; Martinez, A.; Nuel, D. General methodology for the chemoselective N-alkylation of (2,2,6,6)-tetramethylpiperidin-4-ol: Contribution of microwave irradiation. *Tetrahedron Lett.* **2019**, *60*, 240–243, doi:10.1016/j.tetlet.2018.12.020.
6. Vasseur, A.; Membrat, R.; Gatineau, D.; Tenaglia, A.; Nuel, D.; Giordano, L. Secondary Phosphine Oxides as Multitalented Preligands En Route to the Chemoselective Palladium-Catalyzed Oxidation of Alcohols. *ChemCatChem* **2017**, *9*, 728–732, doi:10.1002/cctc.201601261.
7. Vasseur, A.; Membrat, R.; Palpacelli, D.; Giorgi, M.; Nuel, D.; Giordano, L.; Martinez, A. Synthesis of chiral supramolecular bisphosphinite palladacycles through hydrogen transfer-promoted self-assembly process. *Chem. Commun.* **2018**, *54*, 10132–10135, doi:10.1039/C8CC06283H.
8. Frisch, M.J.; Trucks, G.W.; Schlegel, H.B.; Scuseria, G.; Robb, M.; Cheeseman, J.; Scalmani, G.; Barone, V.; Mennucci, B.; Petersson, G.; et al. Gaussian 09, Revision D.01 2013.
